# *HOPS/TMUB1* Enhances Apoptosis in TP53 Mutation-Independent Setting in Human Cancers

**DOI:** 10.3390/ijms25094600

**Published:** 2024-04-23

**Authors:** Nicola Di-Iacovo, Simona Ferracchiato, Stefania Pieroni, Damiano Scopetti, Marilena Castelli, Danilo Piobbico, Luca Pierucci, Marco Gargaro, Davide Chiasserini, Giuseppe Servillo, Maria Agnese Della-Fazia

**Affiliations:** 1 Section of General Pathology, Department of Medicine and Surgery, University of Perugia, 06129 Perugia, Italy; nicola.diiacovo@collaboratori.unipg.it (N.D.-I.); stefania.pieroni@unipg.it (S.P.); damiano.scopetti@collaboratori.unipg.it (D.S.); marilena.castelli@collaboratori.unipg.it (M.C.); danilo.piobbico@collaboratori.unipg.it (D.P.); giuseppe.servillo@unipg.it (G.S.); 2 Section of Biochemical and Health Sciences, Department of Pharmaceutical Sciences, University of Perugia, 06126 Perugia, Italy; marco.gargaro@unipg.it; 3 Section of Physiology and Biochemistry, Department of Medicine and Surgery, University of Perugia, 06129 Perugia, Italy; davide.chiasserini@unipg.it; 4 Centro Universitario di Ricerca sulla Genomica Funzionale (C.U.R.Ge.F.), University of Perugia, 06123 Perugia, Italy

**Keywords:** apoptosis, *HOPS/TMUB1*, p53, gain of function (GOF), TCGA data, breast cancer, lung cancer, pancreatic cancer

## Abstract

*TP53* mutations are prevalent in various cancers, yet the complexity of apoptotic pathway deregulation suggests the involvement of additional factors. *HOPS/TMUB1* is known to extend the half-life of p53 under normal and stress conditions, implying a regulatory function. This study investigates, for the first time, the potential modulatory role of the ubiquitin-like-protein *HOPS/TMUB1* in p53-mutants. A comprehensive analysis of apoptosis in the most frequent p53-mutants, R175, R248, and R273, in SKBR3, MIA PaCa2, and H1975 cells indicates that the overexpression of *HOPS* induces apoptosis at least equivalent to that caused by DNA damage. Immunoprecipitation assays confirm HOPS binding to p53-mutant forms. The interaction of HOPS/TMUB1 with p53-mutants strengthens its effect on the apoptotic cascade, showing a context-dependent gain or loss of function. Gene expression analysis of the *MYC* and *TP63* genes shows that H1975 exhibit a gain-of-function profile, while SKBR3 promote apoptosis in a *TP63*-dependent manner. The TCGA data further corroborate *HOPS/TMUB1*’s positive correlation with apoptotic genes *BAX*, *BBC3*, and *NOXA1*, underscoring its relevance in patient samples. Notably, singular TP53 mutations inadequately explain pathway dysregulation, emphasizing the need to explore additional contributing factors. These findings illuminate the intricate interplay among TP53 mutations, HOPS/TMUB1, and apoptotic pathways, providing valuable insights for targeted cancer interventions.

## 1. Introduction

*TP53* is one of the most important tumor suppressor genes known so far. p53 plays several functions in regulating cell response to stress [1,2]. It works as transcription factor in its homo-tetrameric forms, and in cytoplasm as a monomer. It plays a key role in the onco-suppression mechanism, activating different programs to regulate proliferation, such as cell cycle arrest, apoptosis, and senescence [3,4,5]. Indeed, other vital homeostatic cell programs, antioxidant stress, autophagy, and metabolism regulation have been ascribed to p53 [6,7,8]. Due to the important role covered by p53 in the cell, mutations in this tumor suppressor gene are frequently associated with cancer development. Indeed, more than 50% of all human cancers present mutations in *TP53*, especially in ovarian, colorectal, and small cell lung cancers, while there are clearly fewer in acute myeloid leukemia (AML), thyroid, and bone cancers. Missense mutations represent the main mutational type, with enrichment in pathological samples in the DNA binding domain (DBD) at amino acids R175, R213, R248, R273, and R282. Notably, mutations in a single allele do not guarantee a correct function of p53 in the cell, leading to the loss of the second allele [9]. Different types of *TP53* mutations in various organs can intake different functional behaviors, sometimes assuming an oncogenic characteristic with gain of function (GOF) [10,11,12].

p53 involvement in regulating cell homeostasis requires a high number of post-translational modifications (PTMs), which in turn control *TP53* transcription, stability, and subcellular localization. The E3 ubiquitin ligase *MDM-2* is the most important controller in p53 stability [13]. Recently, other ubiquitin, de-ubiquitinase, and ubiquitin-like proteins have been identified in controlling p53 function, localization, and stability [14,15].

Hepatocyte odd shuttling protein/trans membrane ubiquitin 1 (*HOPS/TMUB1*), (hereafter, HOPS) is a ubiquitin-like (UBL) protein that regulates the stability of several proteins involved in controlling cell proliferation [16]. First identified in liver regeneration, HOPS is a ubiquitous protein that shuttles from the nucleus to the cytoplasm according to the cell cycle phases and different cell stresses, such as genotoxic or oxidative agents or radiation [17]. *HOPS* mRNA is translated into three different proteins with distinct molecular weights. The *HOPS*-knockout mouse model (Hops^−/−^) is alive at birth, even if it shows a slightly reduced Mendelian frequency with respect to Hops^+/+^ mouse [18]. At the cytoplasm, HOPS has a role in mitotic spindle assembly and centrosome duplication, while *HOPS* depletion leads to multipolar spindle and cytokinesis failure both in vitro and in vivo [19]. HOPS is highly expressed in the brain, where it seems to play a role in basal synaptic transmission regulation [20,21].

As a UBL protein, HOPS stabilizes the tumor suppressor p19 and its nucleolar localization by interacting with nucleophosmin (NPM) [22]. In addition, p19Arf stabilized by HOPS results in *TP53* overexpression. HOPS regulates the NF-kB- inflammatory response through the modulation of TRAF6 [23]. Recently, HOPS has been described as an important player in controlling immune checkpoint control, affecting the PD-L1 half-life [24] and prognosis in a number of associated tumors [25,26,27,28,29].

Experiments performed on Hops^−/−^ mice have demonstrated that HOPS not only binds p53 but regulates its half-life. Notably, a lack of *HOPS* decreases p53-dependent apoptosis after DNA damage. HOPS binds p53, protects it from ubiquitination in the cytoplasm, and retains p53 at the mitochondria, determining its accumulation, and in turn, apoptosis execution [18]. Similar results found for Hops^−/+^ show haploinsufficiency in mice with monoallelic deletion [30].

Because HOPS plays an important role in controlling p53 stability, in the present work, we investigated whether HOPS is able to recognize and bind even mutated p53 forms, affecting p53-related transcriptional dependent and independent apoptosis. For this reason, we evaluated the correlation between HOPS and mutated p53 forms in human cancer. Crosschecking the Pan-Cancer Atlas dataset, we selected the three most frequent mutations (R175, R248, and R273) in the p53 sequence, and we chose tumor cell lines with the most frequent mutation of p53 derived from three different organs: breast, pancreas, and lung y. We investigated whether *HOPS* overexpression determines apoptosis and studied the binding of HOPS-p53 in the selected cancer cell lines. Moreover, we explored the possible mechanism and interaction between HOPS and mutated p53 forms (mutp53s) in human cancers.

## 2. Results

### 2.1. DBD Missense Mutations of TP53 per se Are Not Sufficient to Justify the Deregulation of Its Signaling Pathway

**Figure 1:**
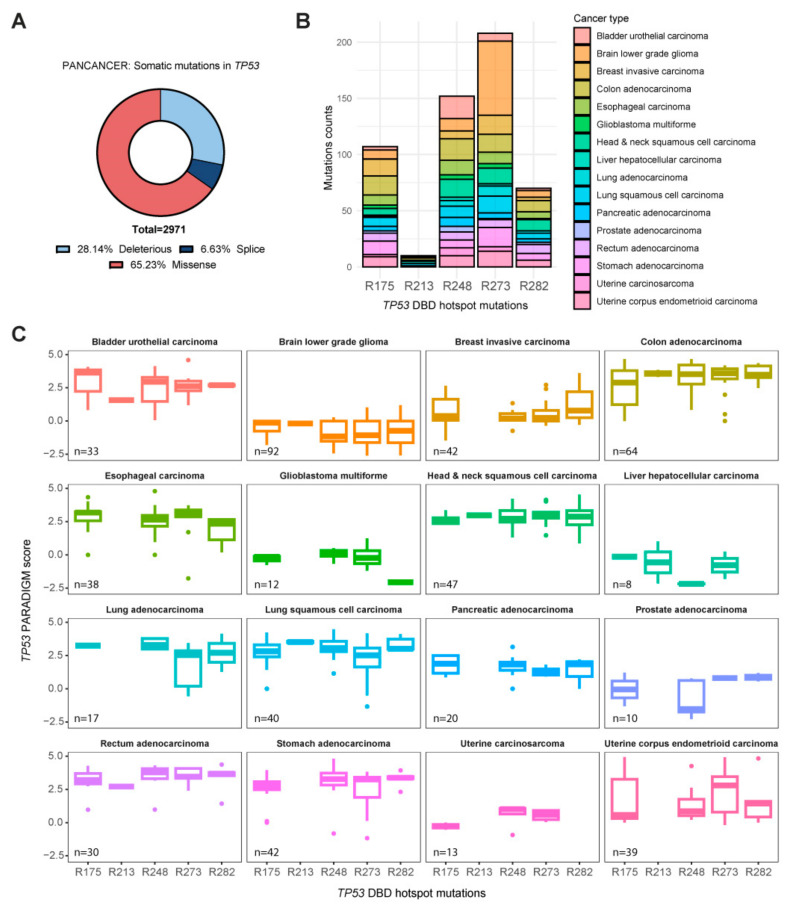
Analysis of patients from the TCGA pan-cancer dataset reporting missense mutations in the DBD of the *TP53* gene. (**A**) Somatic mutation frequency in pan-cancer data. (**B**) Mutation counts in specific arginine of the DBD of p53 (R175, R213, R248, R273, and R282) in selected cancers. (**C**) *TP53* PARADIGM score of patients carrying specific arginine missense mutations in selected cancers. The PARADIGM score is a measure of the activation or inhibition of the most common biological pathways.

Mutation in *TP53* is widely recognized as one of the most common genetic events and determinants of different types of human cancers. An analysis of *TP53* mutations based on The Cancer Genome Atlas (TCGA) dataset provides a detailed overview of the genetic alterations that can impair p53 functions in a broad range of malignancies. Starting with TCGA’s pan-cancer dataset, 2971 patients presenting *TP53* mutations were analyzed. The mutations were classified as deleterious, splice, or missense. From the data, it appears that the majority are missense mutations (65.23%). Deleterious mutations, which are responsible for *TP53* function loss, account for 28.14%. Finally, splice mutations are the least recurrent (6.63%) (Figure 1A).

Intriguingly, the missense mutations have an unpredictable impact on the *TP53* pathway and, consequently, on its function. Indeed, these can cause both a gain and a loss of *TP53* functions. Recurrent *TP53* missense mutations include those affecting the DBD. Specifically, mutations at positions R175, R213, R248, R273, and R282 are known as hotspots and generally cause the loss of p53 binding to DNA. Starting with the pan-cancer dataset, a group of tumors presenting missense mutations in the DBS hotspot sites of p53 was selected (details are given in Section 4). Within the selected tumor dataset, the highest frequency of mutations occurs in the arginine at positions 175, 248, and 273 (Figure 1B). In order to assess the impact of these hotspot mutations within the p53 pathway, data obtained using the PARADIGM algorithm were analyzed [31,32,33,34]. This tool assigns a score based on the integration of pathway, expression, and copy number data to infer the activation of specific features of a pathway that determine its functioning. The PARADIGM score analysis results of the p53 hotspot sites of the selected tumors show that the activation of biological pathways involving *TP53* shows non-specific variation among different tissues. This indicates that missense mutations in the DBD of p53 do not, per se, result in strong alterations in p53 molecular pathways. (Figure 1C). Although the TP53 PARADIGM score is an important pathway tool, it does not guarantee significance in determining the role of *TP53* missense mutations in the pathways in which it is involved. However, if we consider all missense mutations for p53, the sample size increases significantly, confirming our hypothesis. Further confirmation that missense mutations are not sufficient to induce inhibition of the p53 pathway was obtained by comparing all missense mutations with no variant forms in the selected tumors. In the group of tumors analyzed, it appears that the difference between the two subgroups is not significant, except for only three tumor types: invasive breast cancer, colorectal adenocarcinoma, and lung squamous cell carcinoma (Figure S1). The lack of a direct link between *TP53* mutations and alteration of the affected pathway leads to the search for other causes that may induce a loss or gain of p53 function. In this context, the ubiquitin-like modifier HOPS may play a regulatory role in the apoptotic signaling cascade triggered by p53.

### 2.2. HOPS Drives Apoptosis of p53 Mutants

Previous data have shown that the absence of *HOPS* significantly reduces the level of apoptosis in vivo and in vitro, making cells more resistant to apoptosis. Indeed, the ubiquitin-like HOPS driving p53 to the cytoplasm stabilizes p53 as a monomer, prolonging its half-life and ensuring the correct apoptosis activation [18]. Due to the interactions of the two molecules and the high frequency of missense mutations within the p53 DBD, we questioned whether HOPS is able to link to p53 mutants monomers and trigger apoptosis. To this end, three cell lines expressing different *TP53* mutations were selected. SKBR3 is a breast carcinoma cell line expressing a mutation of p53 at R175H; MIA PaCa 2 is derived from pancreatic adenocarcinoma and carries the R248W mutation; finally, the R273H mutation was analyzed in the H1975 lung adenocarcinoma cell line. First, the ability to undergo apoptosis of p53 mutants cell lines was assessed by treatment with etoposide. To test the ability of HOPS to promote apoptosis, assays were performed in which the protein was overexpressed.

**Figure 2:**
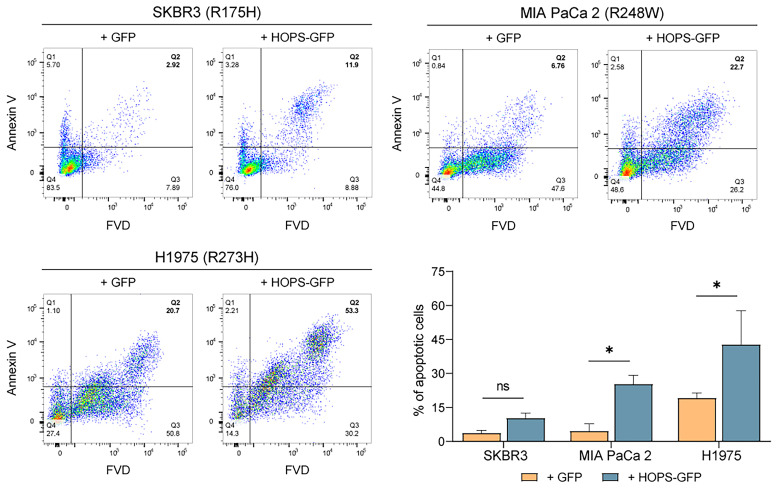
Apoptosis evaluation of SKBR3, MIA PaCa 2, and H1975 cells, carrying different mutations in p53 DBD. The apoptotic cell percentage (Q2) was evaluated in cells transfected with the control vector pEGFP-N1 (+GFP) or in cells overexpressing HOPS (+ HOPS-GFP). The bar graph shows the apoptotic cell percentage, comparing the control and the HOPS overexpression in each cell line analyzed. The data were analyzed using two-way ANOVA with Holm–Sidak’s multiple comparisons test. The values are represented as percentage mean ± SD. * *p* < 0.05, ns *p* > 0.05.

Specifically, H1975, MIA PaCa 2, and SKBR3 cells were transiently transfected with an empty vector (pEGFP-N1) and a HOPS-encoding vector (pEGFP-N1-Hops). The transfected cells (GFP-positive) were selected, and the apoptotic cells were quantified by flow cytometry. Surprisingly, we found that overexpression of HOPS was able to induce apoptosis in the tumor cell lines analyzed. Specifically, in the H1975 and MIA PaCa 2 *HOPS*-overexpressing samples (+HOPS-GFP), the percentage of cells undergoing apoptosis was significantly higher than the related control (+GFP) (Figure 2). Although the three selected cell lines had missense mutations in the DBD of p53, the results obtained by analyzing the apoptotic rate show that apoptotic pathways were still activated. In particular, the overexpression of *HOPS* induced increased cell death regardless of the mutational status of p53.

### 2.3. HOPS Binds TP53 Mutants after Stress Stimuli

**Figure 3:**
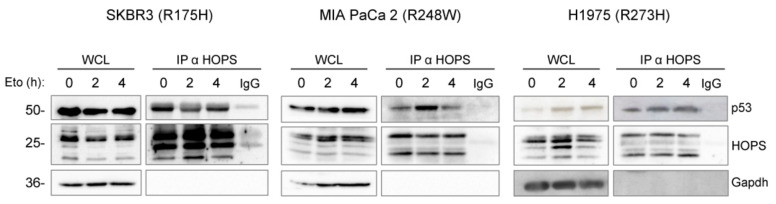
HOPS binds p53 mutants in SKBR3, MIA PaCa 2, and H1975 after etoposide treatment. GAPDH was used as a normalizer in the whole-cell lysate (WCL).

To investigate the ability of HOPS to bind and stabilize mutp53, co-immunoprecipitation (Co-Ip) was performed using cells upon etoposide treatment for different time intervals (0, 2, and 4 h). Notably, in both the untreated and treated cells, HOPS was able to bind the three mutant forms of p53: R175H in SKBR3, R248W in MIA PaCa 2, and R273H in H1975 (Figure 3). These results show that the regulatory role of HOPS on p53 is preserved in mutant cell lines.

### 2.4. HOPS Supports Apoptotic Response in TP53 Mutants

**Figure 4:**
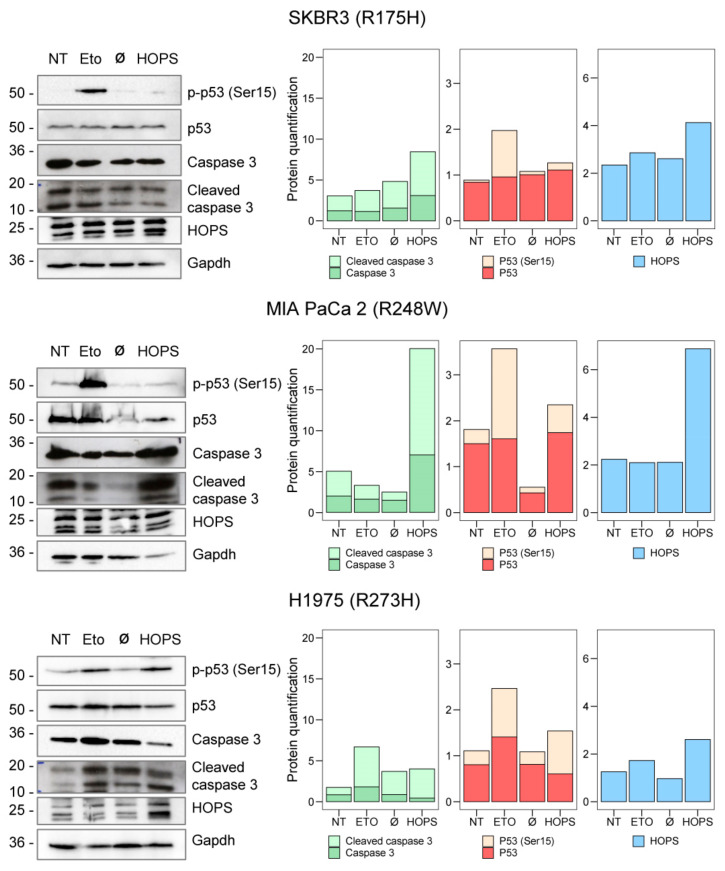
Characterization of apoptosis in SKBR3, MIA PaCa 2, and H1975, after etoposide treatment as a positive control (25 μM etoposide for 4 h) and GFP-tagged HOPS transfection. GAPDH was used as normalizer. The bar graphs are representative of the reported WB and highlight the differences between the total and cleaved caspase 3 (green) and between the total and phosphorylated p53 (red). The HOPS levels are also reported (blue).

Mutp53 has been reported to promote metastasis through different mechanisms, including the phosphorylation of mutp53 at S15, which enhances p53 GOF activities [35]. Since HOPS is able to bind mutp53, we explored whether HOPS has the capacity to stabilize and retain p53 oligomer mutants in the cytoplasm in order to induce apoptosis or drive cancer development. After the transfection of *HOPS* into SKBR3, MIA PaCa 2, and H1975, the protein lysates were analyzed by quantifying the levels of phosphorylated (Ser-15, active form) and unphosphorylated p53. The phosphorylated p53 levels found were compared to those of the etoposide-treated samples, which were used as a positive control. The endogenous p53 levels remained unchanged under the conditions studied. However, in SKBR3 and MIA PaCa 2, the S15 phosphorylated p53 levels were elevated following the etoposide treatment, but not when *HOPS* was overexpressed. Conversely, in H1975, when *HOPS* was overexpressed, phosphorylation at S15 of p53 was almost comparable to that observed after the treatment with etoposide. The assessment of induced apoptosis was performed by quantifying the effector of the apoptotic cascade, cleaved caspase-3. The levels were high in the three cell lines following *HOPS* transfection, confirming that apoptosis was induced regardless of the missense mutations analyzed (Figure 4).

Clearly, the overexpression of *HOPS* can induce apoptosis, possibly depending on the binding that HOPS can establish with different p53 mutants. Indeed, missense mutations that normally cause a loss of p53 function, when kept more stable and functional, as in the case of *HOPS* overexpression, might result in p53 gain of function. *TP53* gain of function can be also estimated by evaluating the gene expression profile, such as the transcriptional activation of *MYC* [36] or impairing *TP63* expression [37]. The transcriptional activation of *MYC* and repression of *TP63* represent the typical gain-of-function profile, as the cell upregulates proliferation by overexpressing *MYC* and inhibits the co-activator activity of *TP63* in apoptosis.

**Figure 5:**
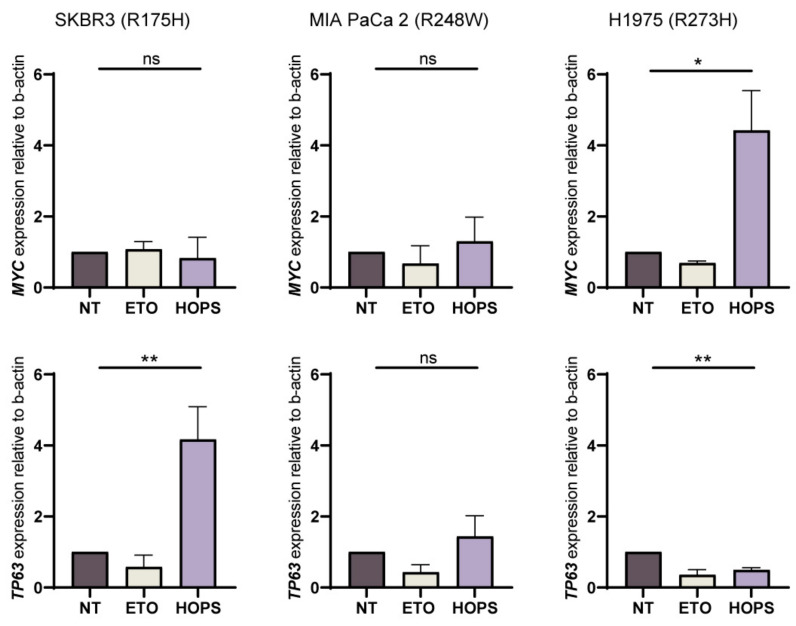
GOF profile identification of SKBR3, MIA PaCa 2, and H1975 by the gene expression evaluation of MYC and TP63. β-actin was used as a normalizer. * *p* < 0.05, ** *p* < 0.01, ns *p* > 0.05.

Although the three cell lines analyzed show an activation of apoptosis, the gene expression analysis shows different profiles. In particular, SKBR3s maintained the same levels of *MYC*, whereas TP63 expression was significantly increased when *HOPS* was overexpressed. This suggests that the observed apoptosis was supported by the co-transactivation of apoptotic genes by *TP63*. Conversely, H1975s show a gain-of-function profile since the onco-suppressor *MYC* was upregulated and *TP63* was repressed when *HOPS* was overexpressed. This confirms what is observed in Figure 4, with the increase in the phosphorylation of p53 at Ser15. In contrast, MIA PaCa 2 does not show a well-defined signature for the genes analyzed (Figure 5).

### 2.5. High Expression Levels of HOPS Positively Correlate with Apoptotic Genes in TCGA Patient Cohort

**Figure 6:**
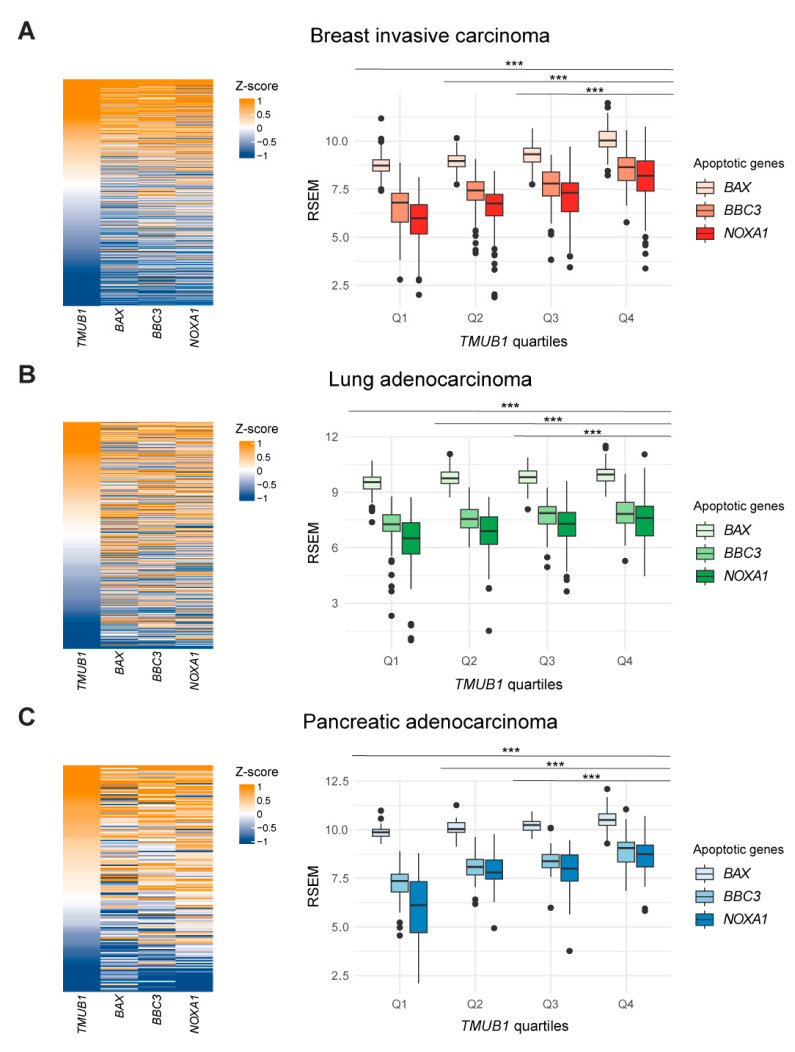
Analysis of TCGA data showing a strong positive correlation between HOPS/TMUB1 and apoptotic gene BAX, BBC3, and NOXA, in breast invasive carcinoma (**A**), lung adenocarcinoma (**B**), and pancreatic adenocarcinoma (**C**). The boxplot displays the correlations in the HOPS/TMUB1 quartile annotations. The gene expression profile was reported as a transformed RSEM normalized count. The calculation of the correlation levels is shown in Figure S2. *** *p* < 0.0001.

To validate the results obtained, gene expression data on breast invasive cancer, lung adenocarcinoma, and pancreatic adenocarcinoma from the TCGA were analyzed. Classical apoptotic genes, which are p53 downstream genes responsible for the pro-apoptotic response *BAX* (BCL2-associated X) and *BBC3* (Bcl-2-binding component 3) were studied. Another gene evaluated is NOXA1 (NADPH oxidase activator 1), which induces the production of ROS (reactive oxygen species), which are responsible for damage induction, and thus, are the activators of apoptosis. Patients were ranked based on their gene expression of *HOPS*. The order obtained was related to the apoptosis genes analyzed and is illustrated in heatmaps. *HOPS* correlates significantly with proapoptotic genes, suggesting an activation of apoptosis in the patients with high *HOPS/TMUB1* expression (Figure S2). However, this correlation becomes more evident and significant as the *HOPS* expression increases, when the patients are divided into four groups according to their quartiles of HOPS expression. Indeed, the fourth quartile (Q4) is significant compared to the previous three quartiles for all three genes analyzed (Figure 6).

It is worth pointing out that annotations of the p53 mutation status are also included in the patient datasets. This does not affect the identified correlation profiles, but rather appears to be a p53-independent phenomenon. Each quartile of *HOPS/TMUB1* analyzed shows a strong positive correlation with *BAX*, *BBC3*, and *NOXA1* compared to the previous quartile, regardless of whether the p53 status is wild-type, mutated, or absent. The correlation values between *HOPS/TMUB1* and the three evaluated genes and their respective significance are shown in Figure S2. The analysis of the available transcriptomic data for invasive breast cancer (19 cell lines), lung adenocarcinoma (79 cell lines), and pancreatic adenocarcinoma (50 cell lines) shows that even in these in vitro cell models, a positive correlation between the expression of *TMUB1* and the genes *BAX*, *BBC3*, and *NOXA1* is observed (Figure S3).

## 3. Discussion

Undoubtedly, the tumor suppressor p53 is one of the main players in cancer control. More than 50% of cancers present mutations in *TP53*, highlighting its critical role in maintaining genomic integrity and preventing cancer development. Here, we provide a comprehensive analysis of p53 missense mutations in the three most frequent arginine “hotspots” of DBD across different tumor types in relation to *HOPS/TMUB1* expression. We demonstrate that it is not individual p53 mutations that lead to over- or under-activity in the p53 pathway, but other factors that may lead to deregulation. These include ubiquitin-like *HOPS/TMUB1*, which is able to lengthen the half-life of p53, prolonging its function. Indeed, in previous studies, we demonstrated that HOPS/TMUB1 not only binds p53, but it is able to protect it from degradation in the cytoplasm, as a monomer. Notably, we showed that even in selected mutant p53 tumor-bearing lines, HOPS/TMUB1 is able to bind the tumor suppressor and determines different functional fates. A detailed analysis of the apoptosis of SKBR3, MIA PaCa 2, and H1975 cells in breast, pancreas, and lung cancer showed that the overexpression of *HOPS* per se is capable of stimulating cell death at levels equal to or higher than those observed after treatment with etoposide. This suggests a potential role of HOPS within apoptotic cascade, where its effect on p53 may enhance or attenuate the final outcome. A classic event involving p53 activity is the initiation of apoptotic cascade in damaged cells. The demonstration that HOPS immunoprecipitates with the p53 mutants analyzed confirms that the apoptotic pathway of p53 can undergo a gain or loss of function depending on the context observed. In particular, gene expression of the onco-suppressor *MYC* and the p53 co-activator *TP63* revealed three different functional profiles. Apoptosis is present in the three lines analyzed, albeit at different rates, but follows different activation pathways. In H1975, the high expression of *MYC*, found when HOPS is overexpressed, might result in a gain of the oncogenic p53 functions. Indeed, on one hand, HOPS might stabilize the p53-R273H mutant under investigation, leading to a deterioration of the system, and culminating in a hyperproliferative phenotype; on the other hand, it is still able to induce an apoptotic response. *HOPS* overexpression in SKBR3 mutated at R175H does not result in increased *MYC*-dependent proliferation but presents an increase in the expression of *TP63*, which supports the apoptotic response and ensures that the pathway still works despite the p53 mutation. Finally, MIA PaCa 2 cells appear to behave differently, confirming the different interactors that may be involved.

The increased *HOPS* expression in cells undergoing apoptosis is also found in patients. In fact, the analyzed TCGA data on breast, lung, and pancreatic cancer report that *HOPS/TMUB1* expression positively correlates with the three genes of the apoptotic response: *BAX*, *BBC3*, and *NOXA1*. The significant correlations found suggest that all four genes are part of the same signaling input, the simultaneous overexpression of which triggers the activation of the apoptotic pathway. It is important to note that this correlation becomes increasingly significant as *TMUB1/HOPS* expression increases. Indeed, the highest quartile of *TMUB1/HOPS* expression (Q4) shows the highest expression of the apoptotic genes analyzed, highlighting an expression-specific trigger. No less important is the consideration of the mutational status of p53 and the expression of *HOPS/TMUB1*. In fact, we found no direct relationship in the gene expression profile of the two genes. The dataset shown in Figure 6 includes both wild-type and mutant p53 patients. Of these, the observed correlation between *HOPS/TMUB1* and the apoptotic genes is independent of the mutational status of p53. This confirms that single mutations in the tumor suppressor gene are neither sufficient nor necessary to manifest deregulation of the pathway in which it is involved. In addition, this positive correlation between the data from in vitro models and the patient data allows us to leverage these cellular models for future insights. These findings illuminate the multifaceted interplay among *TP53* mutations, *HOPS/TMUB1*, and apoptotic pathways, shedding light on potential therapeutic avenues for cancer treatment.

## 4. Materials and Methods

### 4.1. Cell Culture and Treatments

Human breast adenocarcinoma SKBR3 cells (p53 mutated in R175H) were maintained in DMEM medium containing 10% fetal bovine serum (FBS; EuroClone, Milan, Italy), human lung adenocarcinoma H1975 cells (p53 R273H) were cultured in RPMI 1640 medium containing 10% FBS (EuroClone, Milan, Italy), and human pancreatic carcinoma MIA PaCa 2 cells (p53 R248W) were maintained in DMEM medium supplemented with 10% FBS (EuroClone, Milan, Italy), 2.5% horse serum (Thermo Fisher Scientific, Waltham, MA, USA), and 1 mM of sodium pyruvate (Sigma-Aldrich, St. Louis, MO, USA). The cell lines described above were purchased from ATCC. Treatments with etoposide (Sigma-Aldrich) were performed on SKBR3, MIA PaCa 2, and H1975 at 25 μM for the indicated time points. Lipofectamine^®^ LTX (Thermo Fisher Scientific, Waltham, MA, USA) was used to transfect the SKBR3, MIA PaCa 2, and H1975 in accordance with the manufacturer’s instructions.

### 4.2. Cloning and Plasmid

The *HOPS*-coding sequence was amplified and cloned, and its cDNA was subcloned in frames with GFP in the pEGFP-N1 expression vector (Takara Bio USA, San Jose, CA, USA), as previously described [17].

### 4.3. Western Blot, Immunoprecipitation, and Antibodies

Western blot analysis was performed as previously described [17]. Protein extracts were denatured in Laemmli buffer (Tris/HCl at pH 6.8, 200 mM, 8% SDS, 0.4% bromophenol blue, 40% glycerol, and 5% b-mercaptoethanol) and boiled for 5 min at 95 °C. Protein extracts were normalized by SDS–PAGE and Coomassie blue (0.1% blue bromophenol solution, 15% acetic acid, and 25% methanol) staining. The proteins were separated on polyacrylamide gel and transferred by electroblotting onto nitrocellulose membranes (Bio-Rad, Hercules, CA, USA). The membranes were blocked in 5% dry fat-free milk in PBS and probed overnight with the following primary antibodies: anti-p53 (DO-1; Santa Cruz Biotechnology, Dallas, TX, USA), anti-GAPDH (G8795; Sigma-Aldrich), anti p-p53 Ser15 (#9284; Cell Signaling Technology^®^, Danvers, MA, USA), and anti-caspase-3 (#9662; Cell Signaling Technology^®^, Danvers, MA, USA). For the detection of the HOPS protein, a rabbit polyclonal antibody, produced as previously described, was used [18]. The detection was achieved using horseradish-peroxidase-conjugated (HRP) secondary antibody (Bio-Rad) and visualized with ECL (GE Healthcare Life Sciences, Little Chalfont, UK).

For the immunoprecipitation assay, naïve, transfected, or treated cells were lysed in RIPA buffer (50 mM Tris/HCl at pH 8.0, 150 mM NaCl, 0.1% SDS, 1% sodium deoxycholate, 1% Triton X-100, protease inhibitor cocktail (PIC, Sigma-Aldrich, St. Louis, MO, USA), and 1 mM phenylmethylsulphonyl fluoride (PMSF, Sigma-Aldrich, St. Louis, MO, USA). The protein lysates were precleared with protein G or A agarose beads (GE Healthcare Life Sciences, Little Chalfont, UK) for 1 h and then incubated with the specific antibody ON at 4 °C. Normal IgG (Millipore, Darmstadt, Germany) was used as a control. The next day, the immunoprecipitated cells were incubated with protein G or A agarose beads for 2 h at 4 °C. The beads were washed three times in NET gel. The protein was eluted from the beads with Laemmli buffer, boiled for 5 min, and then loaded onto polyacrylamide gels for Western blot analysis, as described above.

### 4.4. Apoptosis Analysis by FACS

The percentages of apoptotic SKBR3, MIA PaCa 2, and H1975 cells were evaluated following transfection and etoposide treatments (25 μM for 4 h) using the eBioscience ^TM^ Annexin V Apoptosis Detection Kit (PEcy7) and fixable viability dye (FVD) EF780 5 × 100T (Thermo Fischer Scientific, Waltham, MA, USA) according to the manufacturer’s instructions. Briefly, the cells were collected by centrifugation, washed with PBS, stained with FVD and Annexin V, respectively, for 30 min at 4 °C and 15 min at room temperature in the dark. The *HOPS*-transfected and positive control cells were gated for GFP (GFP-positive). The samples were run through the LSR Fortessa flow cytometer (BD Biosciences, Franklin Lakes, NJ, USA) and analyzed using FlowJo, (Tree Star, Ashland, OR, USA) analysis software, https://www.flowjo.com/, accessed on 2 March 2024.

### 4.5. RNA Extraction and Real-Time PCR

The total RNA was extracted from the cell lines using NucleoZOL reagent (Macherey-Nagel, Dueren, Germany) according to the manufacturer’s instructions. The cDNA was reverse-transcribed from 1.5 µg of RNA using the iScript cDNA Synthesis kit (Bio-Rad). Real-time PCR was performed with the QuantStudio 3 Real Time PCR System (Applied Biosystem—Thermo Fischer Scientific, Waltham, MA, USA) using the SYBR Green qPCR Master Mix (Applied Biosystems) and ROX as a reference dye. The expressions of all target genes were validated and normalized relative to β-actin expression using the 2^−ΔΔCt^ method. The primers used are listed in Table S1.

### 4.6. TCGA Database and Data Collection

We utilized data from The Cancer Genome Atlas (TCGA) through the UCSC Xena Browser tool provided by the University of California, Santa Cruz (UCSC) (https://xenabrowser.net) (accessed on 5 January 2024) [38]. The UCSC Xena Browser allows users to explore functional genomic datasets to assess correlations between genomic and/or phenotypic variables. The used study datasets were: TCGA pan-cancer (PANCAN), TCGA breast cancer (BRCA), TCGA lung adenocarcinoma (LUAD), and TCGA pancreatic cancer (PAAD). The PANCAN data were selected based on the TP53 somatic mutation type: missense, deleterious, splice, and no variant; and the TP53 PARADIGM score. The selection of the tumor types was made by retaining those with at least two patients with mutations in three of the p53 DBD sites considered (Table S2). The PARADIGM algorithm integrates the pathway, expression, and copy number data to deduce the activation of pathway features within a superimposed pathway network structure, referred to as the SuperPathway. This SuperPathway structure encompasses 1500 constituent pathways sourced from three pathway databases, namely NCI-PID, BioCarta, and Reactome (last updated 05/2013). It incorporates 19,000 pathway features, representing 7369 genes, 9354 complexes, 2092 families, 82 RNAs, 15 miRNAs, and 592 abstract processes. The dataset under consideration comprises the PARADIGM integrated pathway levels (IPLs) of approximately 19,000 pathway features from 33 whitelisted pan-cancer samples within this cohort. These levels were computed using the platform-corrected RNA-seq and GISTIC thresholded gene-level copy number data. The BRCA, LUAD, and PAAD data were filtered based on the TMUB1, BAX, BBC3, and NOXA1 expressions (log2(norm_count + 1) and the TP53 somatic mutation type (as above). The final numbers of patients enrolled in each study are shown in Figure S2.

### 4.7. DepMap Portale and Data Collection

We utilized data from DepMap (https://depmap.org/portal/) (accessed on 2 March 2024), which contains the original Cancer Cell Line Encyclopedia (CCLE) project. The data browser allows for selecting and downloading full data. We selected the cancer cell lines based on the following tumor subtypes: “invasive breast carcinoma”, “lung adenocarcinoma”, and “pancreatic adenocarcinoma”.

### 4.8. Statistics

Statistical analyses were performed using Prism 8.0 (GraphPad) and R-software version 2.7.0 [39]. Each experiment was performed at least three times. The significance test of all the data was analyzed with two-way ANOVA Holm–Sidak’s and Tukey’s multiple-comparison tests. *p*-values of less than 0.05 were considered significant: * *p* < 0.05; ** *p* < 0.01; *** *p* < 0.001.

The used R packages were: ggplot2 [40]; ComplexHeatmap [41,42]; colorRamp2 (package: circlize) [43].

## Data Availability

The data presented in this study are available in the TCGA Hub at https://tcga.xenahubs.net. These data were derived from the following resources available in the public domain: TCGA Pan-Cancer (PANCAN) https://xenabrowser.net/datapages/?cohort=TCGA%20Pan-Cancer%20(PANCAN) (accessed on 2 March 2024); TCGA Breast Cancer (BRCA) https://xenabrowser.net/datapages/?cohort=TCGA%20Breast%20Cancer%20(BRCA) (accessed on 2 March 2024); TCGA Lung Adenocarcinoma (LUAD) https://xenabrowser.net/datapages/?cohort=TCGA%20Lung%20Adenocarcinoma%20(LUAD) (accessed on 2 March 2024); TCGA Pancreatic Cancer (PAAD) at https://xenabrowser.net/datapages/?cohort=TCGA%20Pancreatic%20Cancer%20(PAAD) (accessed on 2 March 2024). The cell lines data analyzed in Figure S3 are available at https://depmap.org/portal/.

## Supplementary Materials

The following supporting information can be downloaded at: https://www.mdpi.com/article/10.3390/ijms25094600/s1.
