# Supplementary material for: HOPS/TMUB1 Enhances Apoptosis in TP53 Mutation-Independent Setting in Human Cancers
Source: Int J Mol Sci. 2024 Apr 23;25(9):4600. doi: 10.3390/ijms25094600 (PMC11083489; doi:10.3390/ijms25094600)
Supplement: Supplementary file 1 [file ijms-25-04600-s001.zip › FACS raw data.pdf]

FACS analysis raw data

Samples identification:

SAMPLE 1) Control

SAMPLE 2) Dead cells (FVD)

SAMPLE 3) FVD control

SAMPLE 4) Cy7 control

SAMPLE 5) GFP control

SAMPLE 6) GFP + FVD control

SAMPLE 7) GFP + Cy7 control

SAMPLE 8) pEGFP-N1 (+GFP)

SAMPLE 9) pEGFP-N1-HOPS (+HOPS-GFP)

SKBR3

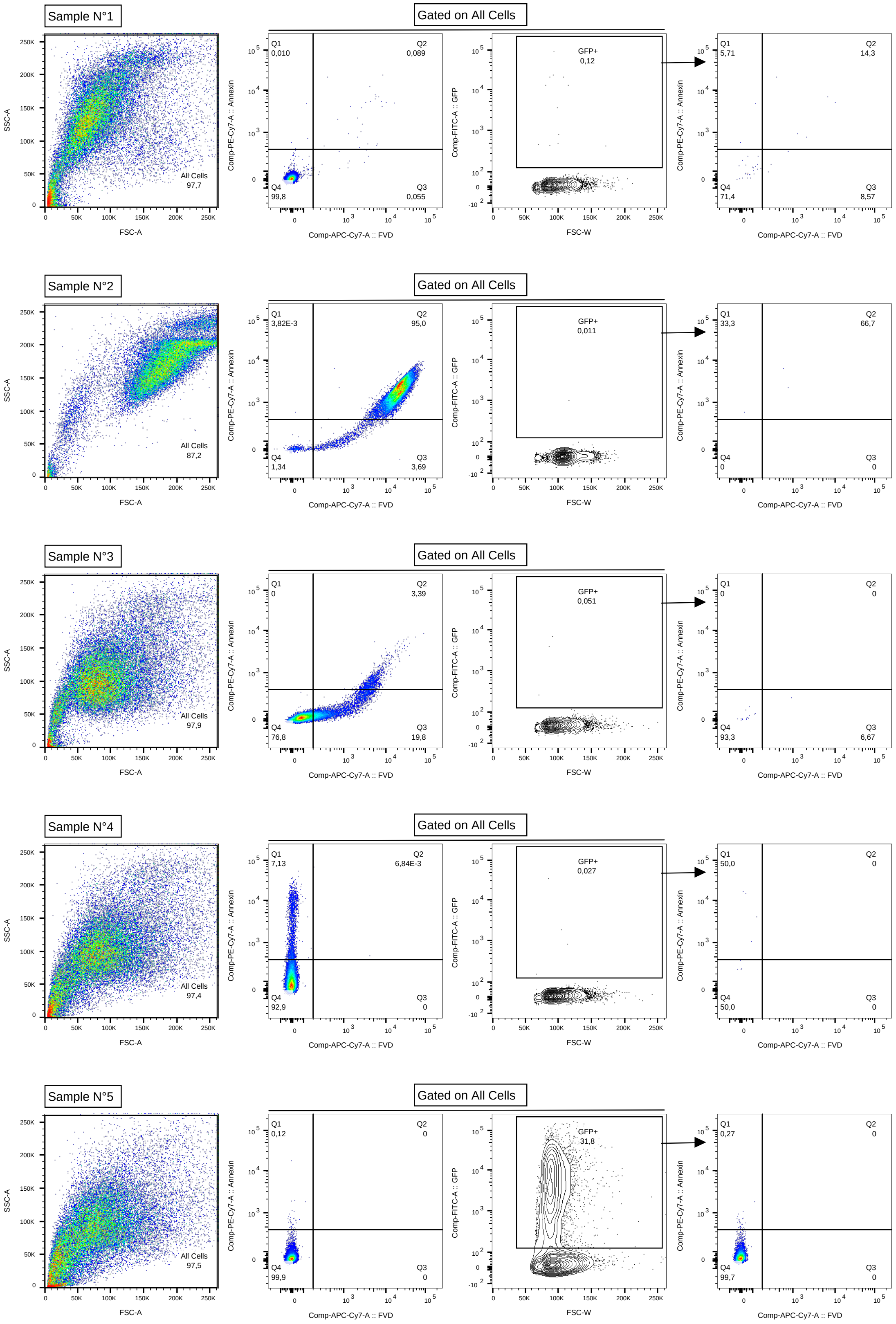
Sample N°1
Gated on All Cells
All Cells 97,7
Q1 0,010
Q2 0,089
Q3 0,055
Q4 99,8
GFP+ 0,12
Q1 5,71
Q2 14,3
Q3 8,57
Q4 71,4
Sample N°2
Gated on All Cells
All Cells 87,2
Q1 3,82E-3
Q2 95,0
Q3 3,69
Q4 1,34
GFP+ 0,011
Q1 33,3
Q2 66,7
Q3 0
Q4 0
Sample N°3
Gated on All Cells
All Cells 97,9
Q1 0
Q2 3,39
Q3 19,8
Q4 76,8
GFP+ 0,051
Q1 0
Q2 0
Q3 6,67
Q4 93,3
Sample N°4
Gated on All Cells
All Cells 97,4
Q1 7,13
Q2 6,84E-3
Q3 0
Q4 92,9
GFP+ 0,027
Q1 50,0
Q2 0
Q3 0
Q4 50,0
Sample N°5
Gated on All Cells
All Cells 97,5
Q1 0,12
Q2 0
Q3 0
Q4 99,9
GFP+ 31,8
Q1 0,27
Q2 0
Q3 0
Q4 99,7
SSC-A
FSC-A
FSC-W
Comp-PE-Cy7-A :: Annexin
Comp-APC-Cy7-A :: FVD
Comp-FITC-A :: GFP

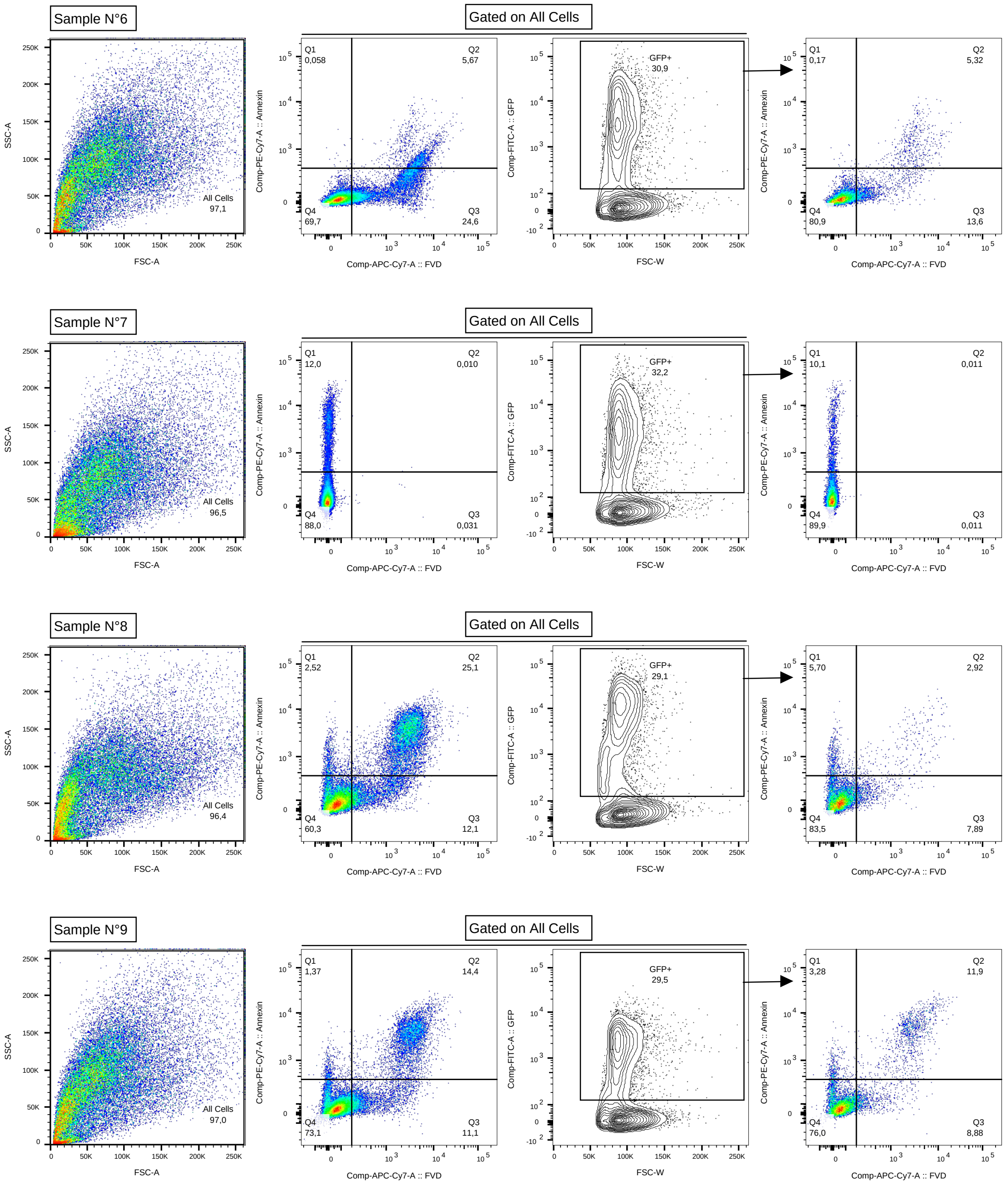

Sample N°6
Gated on All Cells
All Cells 97,1
Q1 0,058
Q2 5,67
Q3 24,6
Q4 69,7
GFP+ 30,9
Q1 0,17
Q2 5,32
Q3 13,6
Q4 80,9
Sample N°7
Gated on All Cells
All Cells 96,5
Q1 12,0
Q2 0,010
Q3 0,031
Q4 88,0
GFP+ 32,2
Q1 10,1
Q2 0,011
Q3 0,011
Q4 89,9
Sample N°8
Gated on All Cells
All Cells 96,4
Q1 2,52
Q2 25,1
Q3 12,1
Q4 60,3
GFP+ 29,1
Q1 5,70
Q2 2,92
Q3 7,89
Q4 83,5
Sample N°9
Gated on All Cells
All Cells 97,0
Q1 1,37
Q2 14,4
Q3 11,1
Q4 73,1
GFP+ 29,5
Q1 3,28
Q2 11,9
Q3 8,88
Q4 76,0
SSC-A
FSC-A
Comp-PE-Cy7-A :: Annexin
Comp-APC-Cy7-A :: FVD
Comp-FITC-A :: GFP
FSC-W

MIA PaCa 2

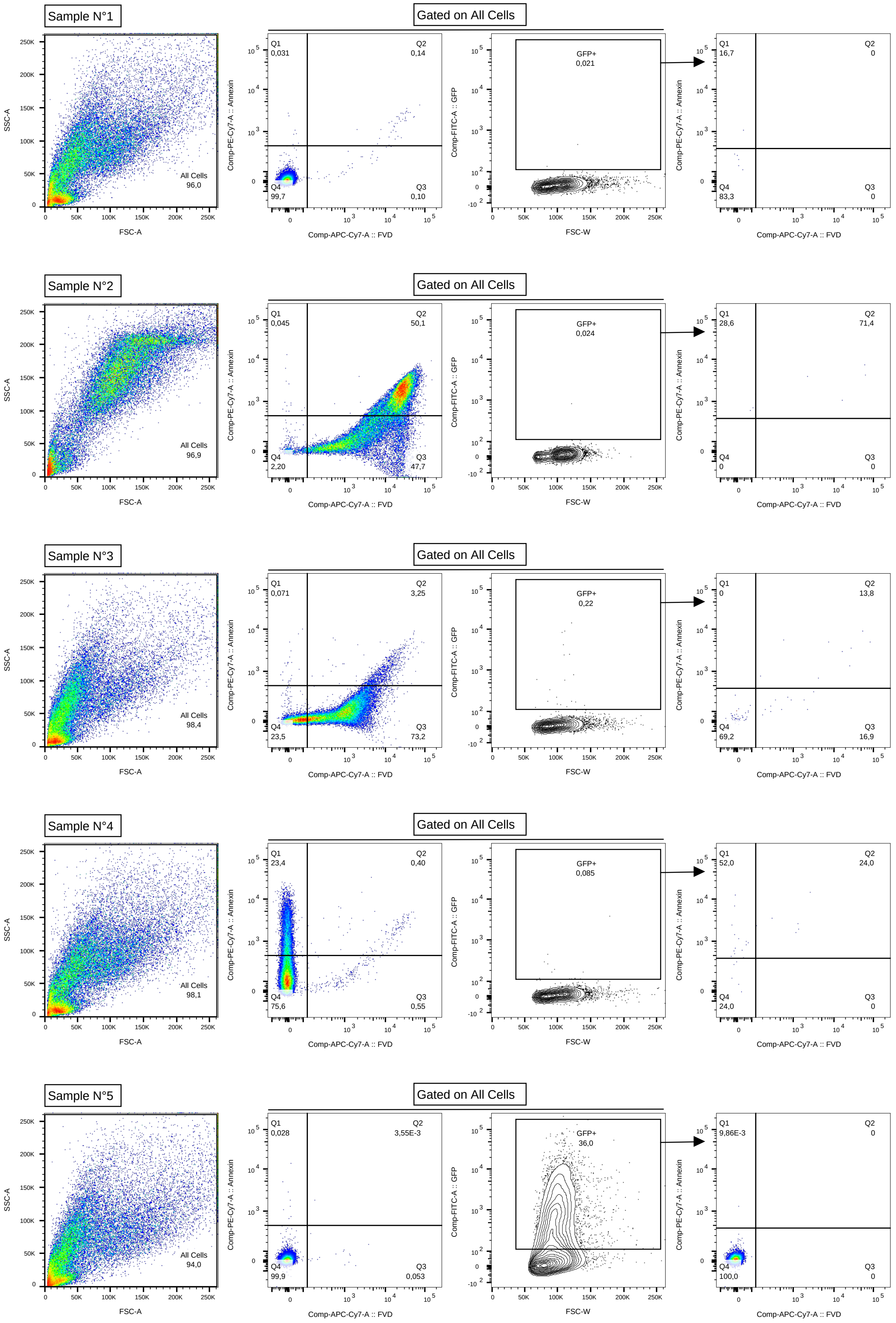
Sample N°1
Gated on All Cells
All Cells 96,0
Q1 0,031
Q2 0,14
Q3 0,10
Q4 99,7
GFP+ 0,021
Q1 16,7
Q2 0
Q3 0
Q4 83,3
Sample N°2
Gated on All Cells
All Cells 96,9
Q1 0,045
Q2 50,1
Q3 47,7
Q4 2,20
GFP+ 0,024
Q1 28,6
Q2 71,4
Q3 0
Q4 0
Sample N°3
Gated on All Cells
All Cells 98,4
Q1 0,071
Q2 3,25
Q3 73,2
Q4 23,5
GFP+ 0,22
Q1 0
Q2 13,8
Q3 16,9
Q4 69,2
Sample N°4
Gated on All Cells
All Cells 98,1
Q1 23,4
Q2 0,40
Q3 0,55
Q4 75,6
GFP+ 0,085
Q1 52,0
Q2 24,0
Q3 0
Q4 24,0
Sample N°5
Gated on All Cells
All Cells 94,0
Q1 0,028
Q2 3,55E-3
Q3 0,053
Q4 99,9
GFP+ 36,0
Q1 9,86E-3
Q2 0
Q3 0
Q4 100,0
SSC-A
FSC-A
Comp-PE-Cy7-A :: Annexin
Comp-APC-Cy7-A :: FVD
Comp-FITC-A :: GFP
FSC-W

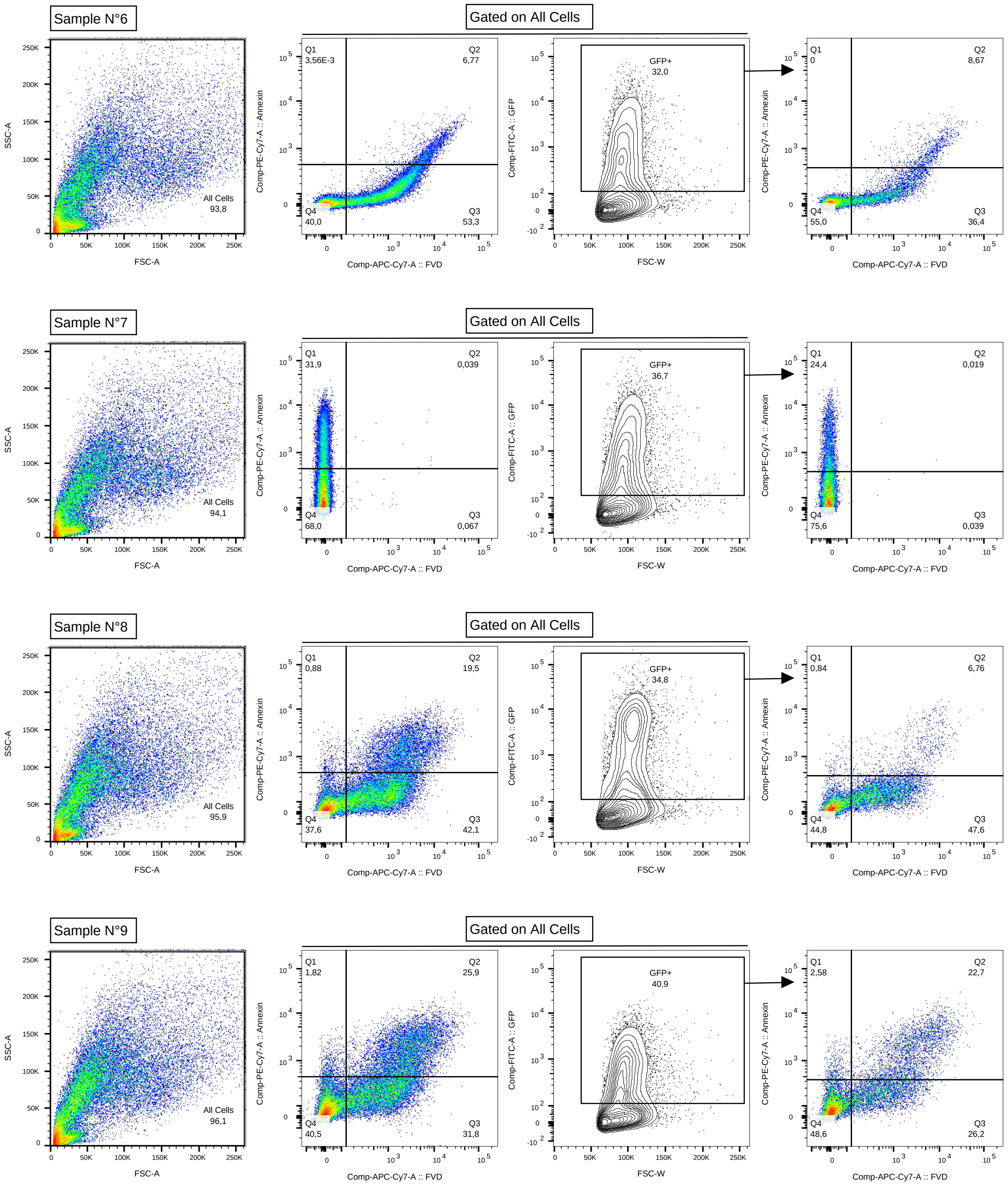

Sample N°6
Gated on All Cells
SSC-A
FSC-A
All Cells
93,8
Comp-PE-Cy7-A :: Annexin
Comp-APC-Cy7-A :: FVD
Q1
3,56E-3
Q2
6,77
Q3
53,3
Q4
40,0
Comp-FITC-A :: GFP
FSC-W
GFP+
32,0
Q1
0
Q2
8,67
Q3
36,4
Q4
55,0
Sample N°7
Gated on All Cells
All Cells
94,1
Q1
31,9
Q2
0,039
Q3
0,067
Q4
68,0
GFP+
36,7
Q1
24,4
Q2
0,019
Q3
0,039
Q4
75,6
Sample N°8
Gated on All Cells
All Cells
95,9
Q1
0,88
Q2
19,5
Q3
42,1
Q4
37,6
GFP+
34,8
Q1
0,84
Q2
6,76
Q3
47,6
Q4
44,8
Sample N°9
Gated on All Cells
All Cells
96,1
Q1
1,82
Q2
25,9
Q3
31,8
Q4
40,5
GFP+
40,9
Q1
2,58
Q2
22,7
Q3
26,2
Q4
48,6

H1975

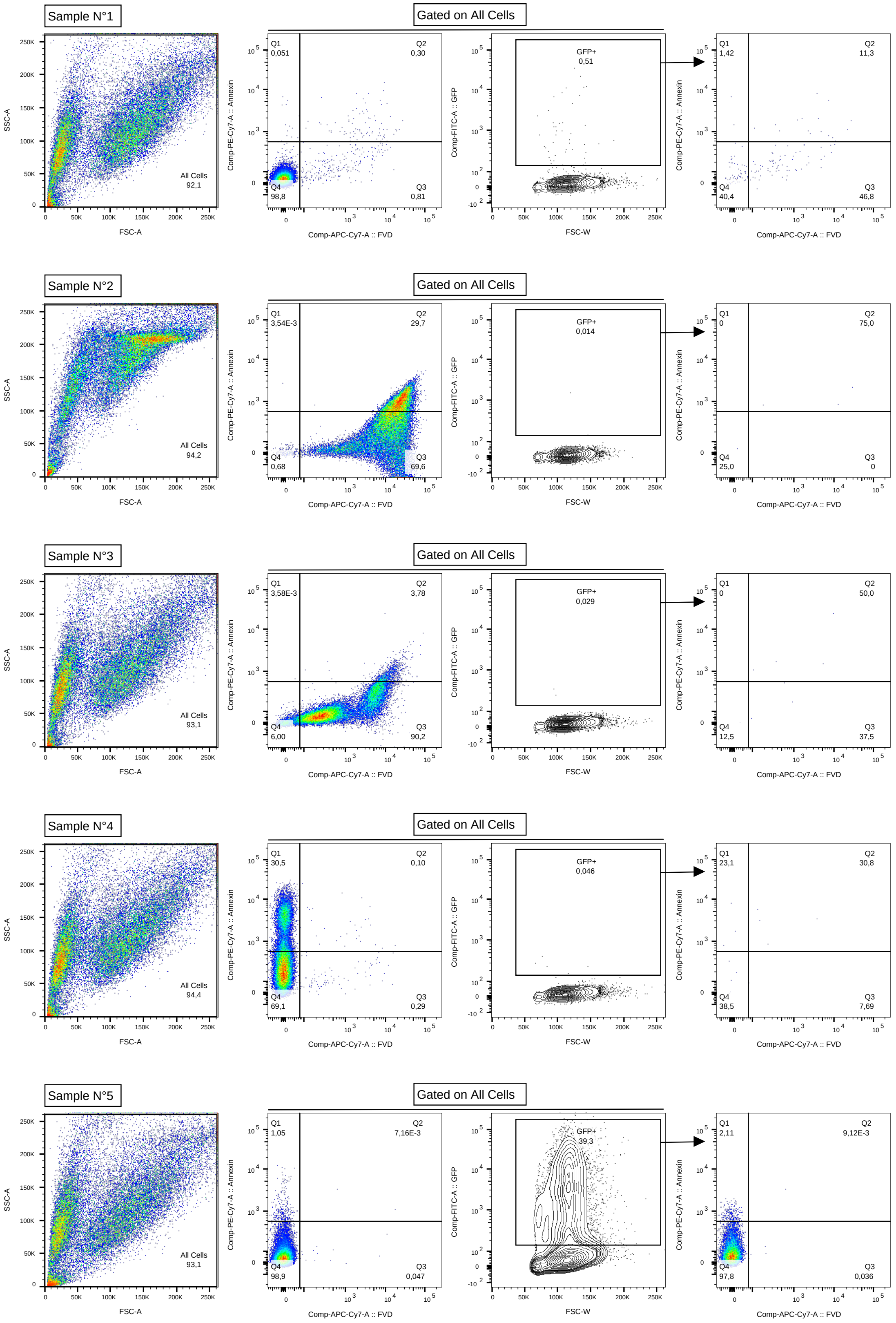

Sample N°1
Gated on All Cells
All Cells 92,1
Q1 0,051
Q2 0,30
Q3 0,81
Q4 98,8
GFP+ 0,51
Q1 1,42
Q2 11,3
Q3 46,8
Q4 40,4
Sample N°2
Gated on All Cells
All Cells 94,2
Q1 3,54E-3
Q2 29,7
Q3 69,6
Q4 0,68
GFP+ 0,014
Q1 0
Q2 75,0
Q3 0
Q4 25,0
Sample N°3
Gated on All Cells
All Cells 93,1
Q1 3,58E-3
Q2 3,78
Q3 90,2
Q4 6,00
GFP+ 0,029
Q1 0
Q2 50,0
Q3 37,5
Q4 12,5
Sample N°4
Gated on All Cells
All Cells 94,4
Q1 30,5
Q2 0,10
Q3 0,29
Q4 69,1
GFP+ 0,046
Q1 23,1
Q2 30,8
Q3 7,69
Q4 38,5
Sample N°5
Gated on All Cells
All Cells 93,1
Q1 1,05
Q2 7,16E-3
Q3 0,047
Q4 98,9
GFP+ 39,3
Q1 2,11
Q2 9,12E-3
Q3 0,036
Q4 97,8
SSC-A
FSC-A
Comp-PE-Cy7-A :: Annexin
Comp-APC-Cy7-A :: FVD
Comp-FITC-A :: GFP
FSC-W

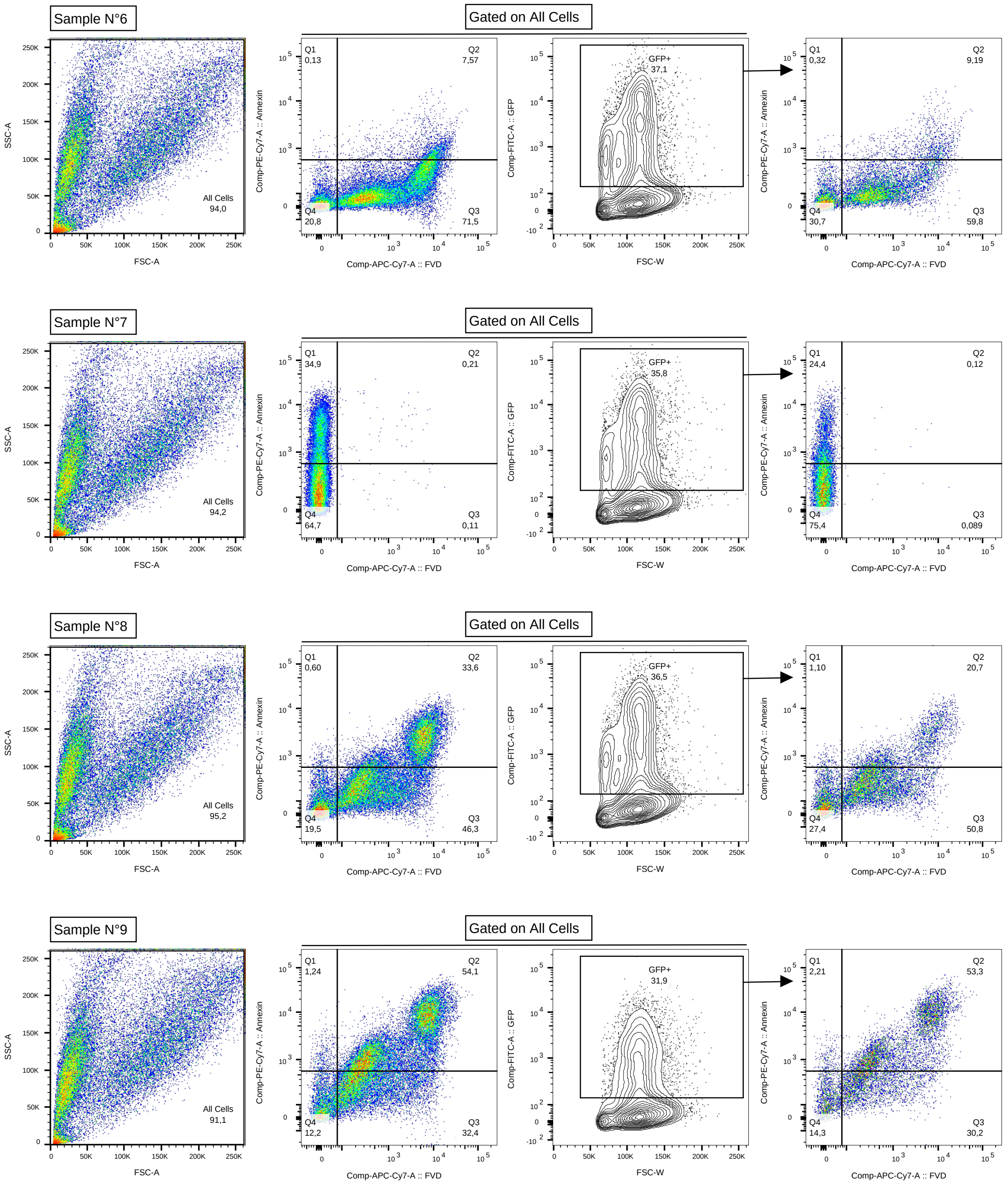

Sample N°6
Gated on All Cells
SSC-A
FSC-A
All Cells 94,0
Comp-PE-Cy7-A :: Annexin
Comp-APC-Cy7-A :: FVD
Q1 0,13
Q2 7,57
Q3 71,5
Q4 20,8
Comp-FITC-A :: GFP
FSC-W
GFP+ 37,1
Q1 0,32
Q2 9,19
Q3 59,8
Q4 30,7
Sample N°7
Gated on All Cells
All Cells 94,2
Q1 34,9
Q2 0,21
Q3 0,11
Q4 64,7
GFP+ 35,8
Q1 24,4
Q2 0,12
Q3 0,089
Q4 75,4
Sample N°8
Gated on All Cells
All Cells 95,2
Q1 0,60
Q2 33,6
Q3 46,3
Q4 19,5
GFP+ 36,5
Q1 1,10
Q2 20,7
Q3 50,8
Q4 27,4
Sample N°9
Gated on All Cells
All Cells 91,1
Q1 1,24
Q2 54,1
Q3 32,4
Q4 12,2
GFP+ 31,9
Q1 2,21
Q2 53,3
Q3 30,2
Q4 14,3
