# Supplementary material for: HOPS/TMUB1 Enhances Apoptosis in TP53 Mutation-Independent Setting in Human Cancers
Source: Int J Mol Sci. 2024 Apr 23;25(9):4600. doi: 10.3390/ijms25094600 (PMC11083489; doi:10.3390/ijms25094600)
Supplement: Supplementary file 1 [file ijms-25-04600-s001.zip › Figure S1 with caption.pdf]

Figure S1

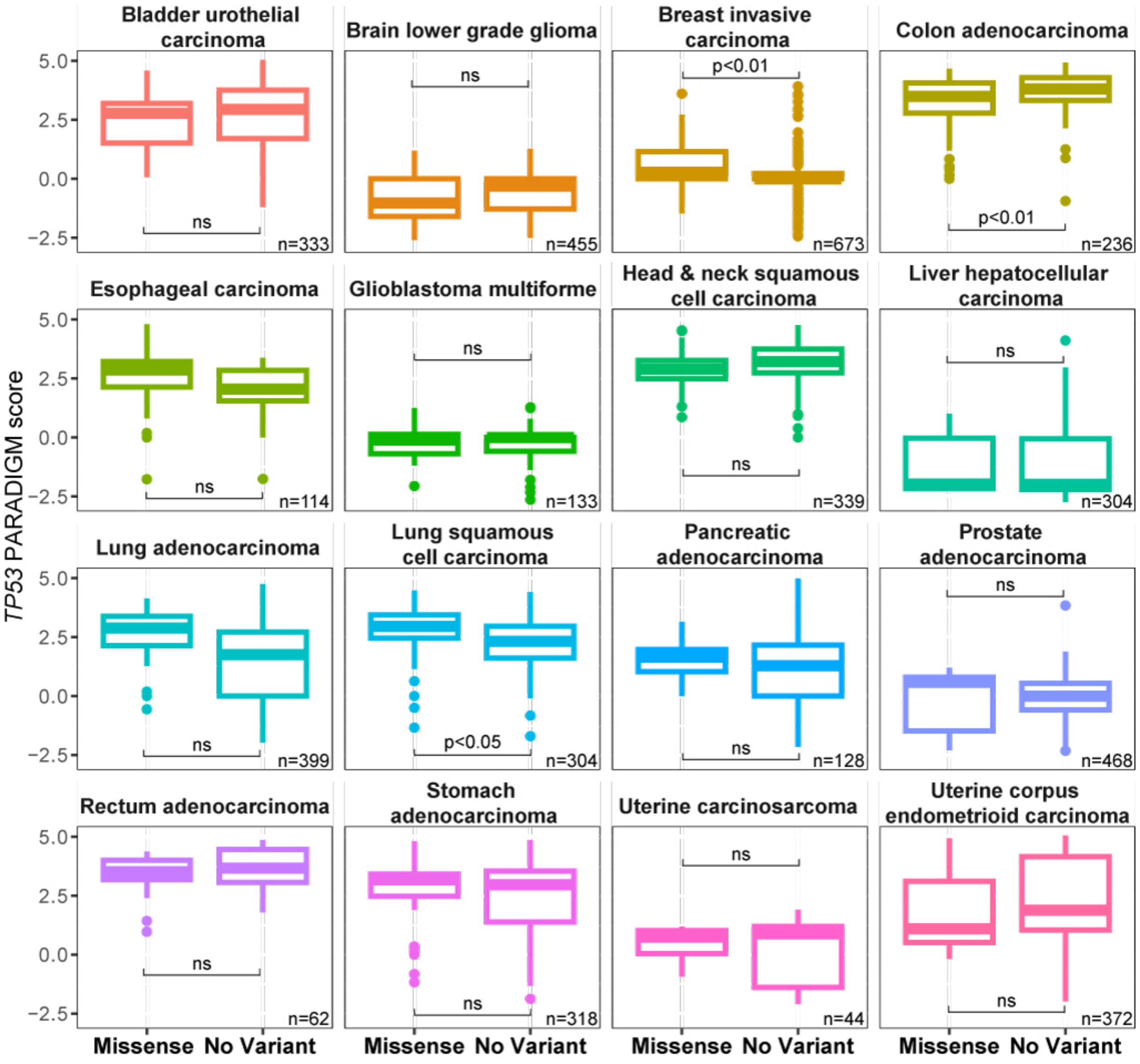

Figure S1. TP53 PARADIGM score in selected tumors comparing two subgroups: p53 missense mutations versus no variant.
