# Supplementary material for: HOPS/TMUB1 Enhances Apoptosis in TP53 Mutation-Independent Setting in Human Cancers
Source: Int J Mol Sci. 2024 Apr 23;25(9):4600. doi: 10.3390/ijms25094600 (PMC11083489; doi:10.3390/ijms25094600)
Supplement: Supplementary file 1 [file ijms-25-04600-s001.zip › Figure S2 with caption.pdf]

Figure S2

Breast invasive carcinoma: 1067 patients

Genes correlated	Pearson's rho (r)	P-value
TMUB1-BAX	0,67	$5,56e^{-141}$
TMUB1-BBC3	0,59	$1,69e^{-100}$
TMUB1-NOXA1	0,57	$5,28e^{-91}$

Heatmap correlation index

	BAX	BBC3	NOXA1
2-1	*** p <0.001	*** p <0.001	*** p <0.001
3-1	*** p <0.001	*** p <0.001	*** p <0.001
4-1	*** p <0.001	*** p <0.001	*** p <0.001
3-2	*** p <0.001	*** p <0.001	*** p <0.001
4-2	*** p <0.001	*** p <0.001	*** p <0.001
4-3	*** p <0.001	*** p <0.001	*** p <0.001

Quartiles comparison

Lung adenocarcinoma: 475 patients

Genes correlated	Pearson's rho (r)	P-value
TMUB1-BAX	0,41	$5,23e^{-21}$
TMUB1-BBC3	0,46	$4,98e^{-26}$
TMUB1-NOXA1	0,43	$9,88e^{-23}$

Heatmap correlation index

	BAX	BBC3	NOXA1
2-1	*** p <0.001	*** p <0.001	** p =0.008
3-1	*** p <0.001	*** p <0.001	*** p <0.001
4-1	*** p <0.001	*** p <0.001	*** p <0.001
3-2	ns p =0.945	ns p =0.473	ns p =0.101
4-2	* p =0.032	* p =0.014	*** p <0.001
4-3	ns p =0.130	ns p =0.394	ns p =0.187

Quartiles comparison

Pancreatic adenocarcinoma: 164 patients

Genes correlated	Pearson's rho (r)	P-value
TMUB1-BAX	0,51	$2,49e^{-12}$
TMUB1-BBC3	0,72	$2,47e^{-22}$
TMUB1-NOXA1	0,75	$8,25e^{-32}$

Heatmap correlation index

	BAX	BBC3	NOXA1
2-1	ns p =0.104	*** p <0.001	*** p <0.001
3-1	*** p <0.001	*** p <0.001	*** p <0.001
4-1	*** p <0.001	*** p <0.001	*** p <0.001
3-2	ns p =0.422	ns p =0.316	ns p =0.999
4-2	*** p <0.001	*** p <0.001	* p =0.034
4-3	* p =0.019	* p =0.014	* p =0.037

Quartiles comparison

Figure S2. Correlation values related to heatmap showed in Figure 6 into selected tumors data. The tables on the left display the p-value of comparison in HOPS/TMUB1 quartiles among the apoptotic genes BAX, BBC3 and NOXA1.

Table 1. Primer sequences used in the RT-qPCR assays.

**Target**	**Primer name**	**Sequence (5'→3')**
β-*actin*	β-actin H F<br>β-actin H R	CACTCTTCCAGCCTTCCT<br>CAGGTCTTTGCGGATGTC
Myc	Myc H F<br>Myc H R	CACACATCAGCACAACTACG<br>CTGTCCAACTTGACCCTCTT
Tp63	TP63 H F<br>TP63 H R	CGTGTCCTTCCAGCAGTC<br>GGCAGTAGAGTTTCTTCAGTTCA
