# Supplementary material for: HOPS/TMUB1 Enhances Apoptosis in TP53 Mutation-Independent Setting in Human Cancers
Source: Int J Mol Sci. 2024 Apr 23;25(9):4600. doi: 10.3390/ijms25094600 (PMC11083489; doi:10.3390/ijms25094600)
Supplement: Supplementary file 1 [file ijms-25-04600-s001.zip › Figure S3 with caption.pdf]

Figure S3

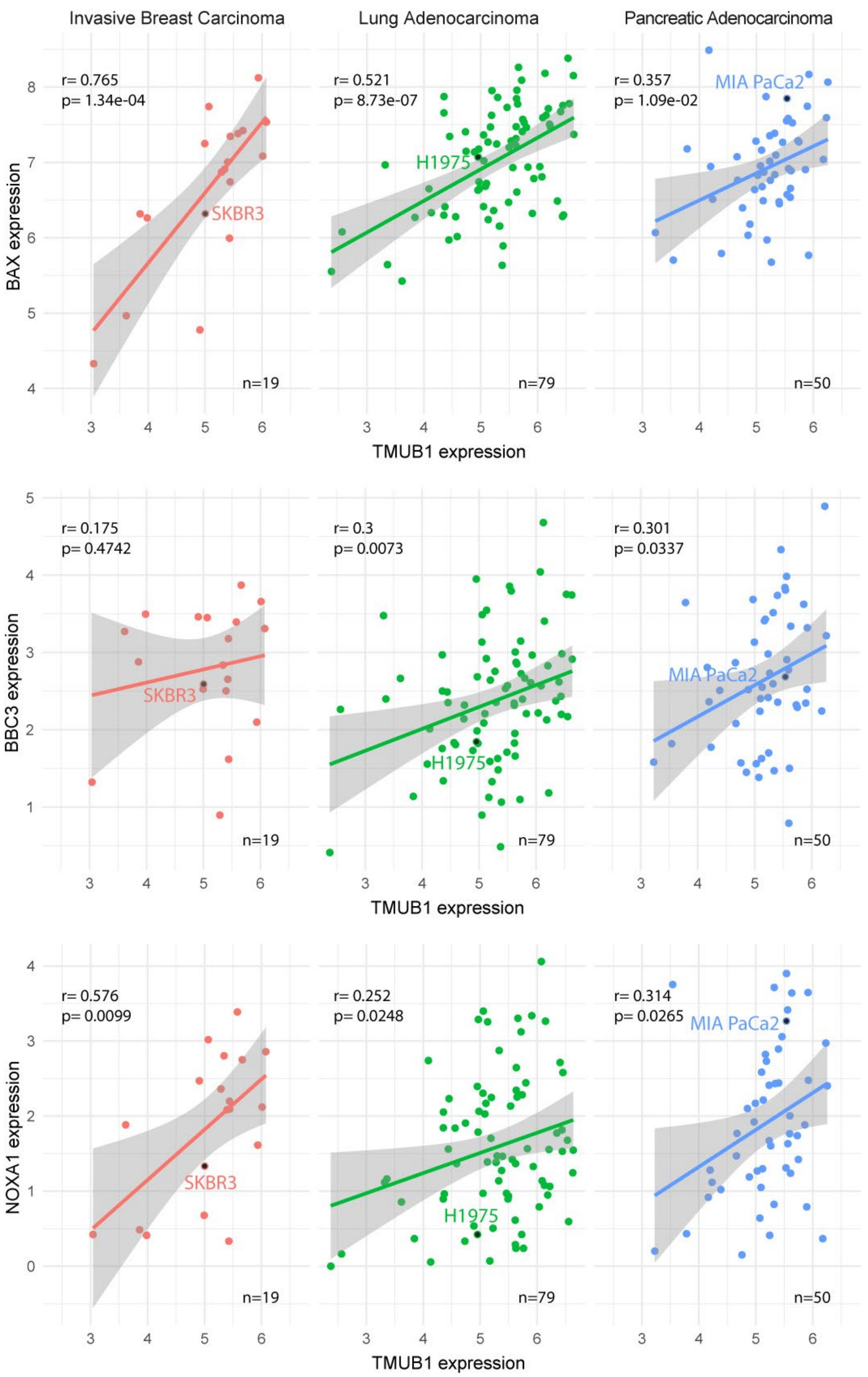

Figure S3. Correlation analysis in selected cell lines type (Invasive Breast Carcinoma, n=19; Lung Adenocarcinoma, n=79; Pancreatic Adenocarcinoma, n=50) between *TMUB1* expression and the apoptotic genes analysed (*BAX*, *BBC3* and *NOXA1*). Correlation score is reported in each scatter plot. Regression line is shown to estimate the linear relationship between the genes.
