# Supplementary material for: HOPS/TMUB1 Enhances Apoptosis in TP53 Mutation-Independent Setting in Human Cancers
Source: Int J Mol Sci. 2024 Apr 23;25(9):4600. doi: 10.3390/ijms25094600 (PMC11083489; doi:10.3390/ijms25094600)
Supplement: Supplementary file 1 [file ijms-25-04600-s001.zip › Supplementary information.pdf]

Figure S1

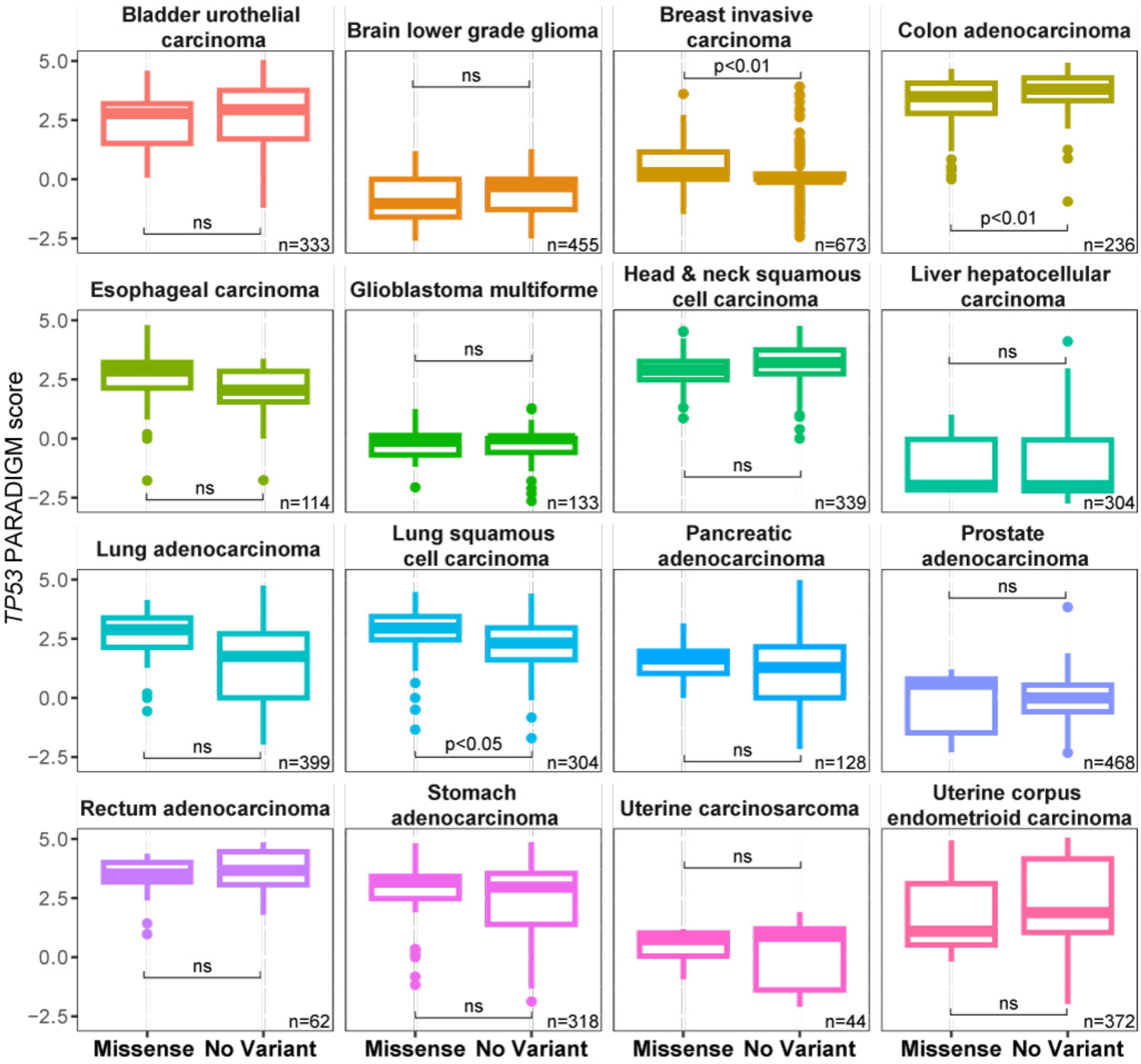

Figure S1. TP53 PARADIGM score in selected tumors comparing two subgroups: p53 missense mutations versus no variant.

Figure S2

Breast invasive carcinoma: 1067 patients

Genes correlated	Pearson's rho (r)	P-value
TMUB1-BAX	0,67	$5{,}56e^{-141}$
TMUB1-BBC3	0,59	$1{,}69e^{-100}$
TMUB1-NOXA1	0,57	$5{,}28e^{-91}$

Heatmap correlation index

	BAX	BBC3	NOXA1
2-1	*** p <0.001	*** p <0.001	*** p <0.001
3-1	*** p <0.001	*** p <0.001	*** p <0.001
4-1	*** p <0.001	*** p <0.001	*** p <0.001
3-2	*** p <0.001	*** p <0.001	*** p <0.001
4-2	*** p <0.001	*** p <0.001	*** p <0.001
4-3	*** p <0.001	*** p <0.001	*** p <0.001

Quartiles comparison

Lung adenocarcinoma: 475 patients

Genes correlated	Pearson's rho (r)	P-value
TMUB1-BAX	0,41	$5{,}23e^{-21}$
TMUB1-BBC3	0,46	$4{,}98e^{-26}$
TMUB1-NOXA1	0,43	$9{,}88e^{-23}$

Heatmap correlation index

	BAX	BBC3	NOXA1
2-1	*** p <0.001	*** p <0.001	** p =0.008
3-1	*** p <0.001	*** p <0.001	*** p <0.001
4-1	*** p <0.001	*** p <0.001	*** p <0.001
3-2	ns p =0.945	ns p =0.473	ns p =0.101
4-2	* p =0.032	* p =0.014	*** p <0.001
4-3	ns p =0.130	ns p =0.394	ns p =0.187

Quartiles comparison

Pancreatic adenocarcinoma: 164 patients

Genes correlated	Pearson's rho (r)	P-value
TMUB1-BAX	0,51	$2{,}49e^{-12}$
TMUB1-BBC3	0,72	$2{,}47e^{-22}$
TMUB1-NOXA1	0,75	$8{,}25e^{-32}$

Heatmap correlation index

	BAX	BBC3	NOXA1
2-1	ns p =0.104	*** p <0.001	*** p <0.001
3-1	*** p <0.001	*** p <0.001	*** p <0.001
4-1	*** p <0.001	*** p <0.001	*** p <0.001
3-2	ns p =0.422	ns p =0.316	ns p =0.999
4-2	*** p <0.001	*** p <0.001	* p =0.034
4-3	* p =0.019	* p =0.014	* p =0.037

Quartiles comparison

Figure S2. Correlation values related to heatmap showed in Figure 6 into selected tumors data. The tables on the left display the p-value of comparison in HOPS/TMUB1 quartiles among the apoptotic genes BAX, BBC3 and NOXA1.

Table 1. Primer sequences used in the RT-qPCR assays.

**Target**	**Primer name**	**Sequence (5'→3')**
β-*actin*	β-actin H F<br>β-actin H R	CACTCTTCCAGCCTTCCT<br>CAGGTCTTTGCGGATGTC
Myc	Myc H F<br>Myc H R	CACACATCAGCACAACTACG<br>CTGTCCAACTTGACCCTCTT
Tp63	TP63 H F<br>TP63 H R	CGTGTCCTTCCAGCAGTC<br>GGCAGTAGAGTTTCTTCAGTTCA

Figure S3

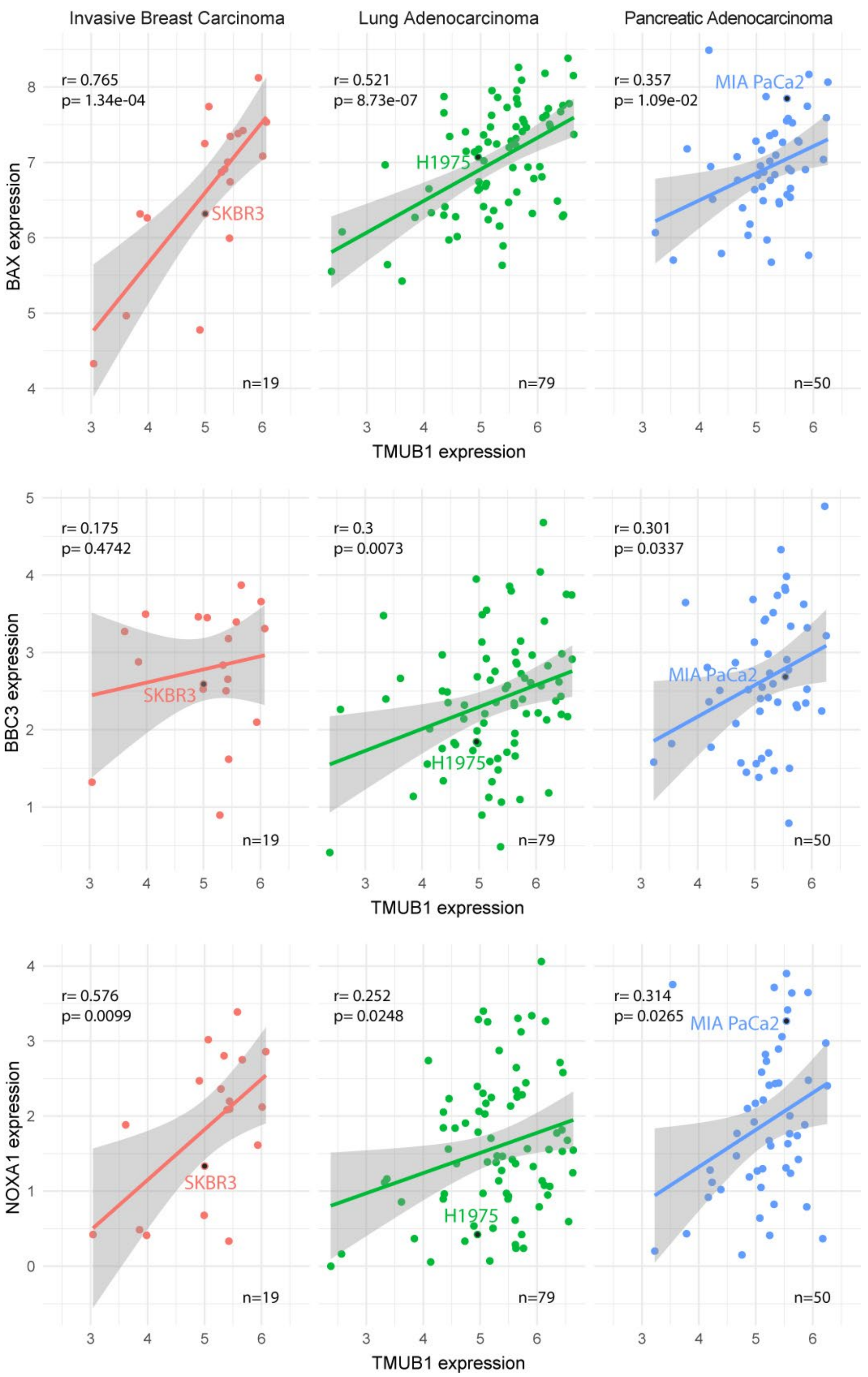

Figure S3. Correlation analysis in selected cell lines type (Invasive Breast Carcinoma, n=19; Lung Adenocarcinoma, n=79; Pancreatic Adenocarcinoma, n=50) between *TMUB1* expression and the apoptotic genes analysed (*BAX*, *BBC3* and *NOXA1*). Correlation score is reported in each scatter plot. Regression line is shown to estimate the linear relationship between the genes.

FACS analysis raw data

Samples identification:

SAMPLE 1) Control

SAMPLE 2) Dead cells (FVD)

SAMPLE 3) FVD control

SAMPLE 4) Cy7 control

SAMPLE 5) GFP control

SAMPLE 6) GFP + FVD control

SAMPLE 7) GFP + Cy7 control

SAMPLE 8) pEGFP-N1 (+GFP)

SAMPLE 9) pEGFP-N1-HOPS (+HOPS-GFP)

SKBR3

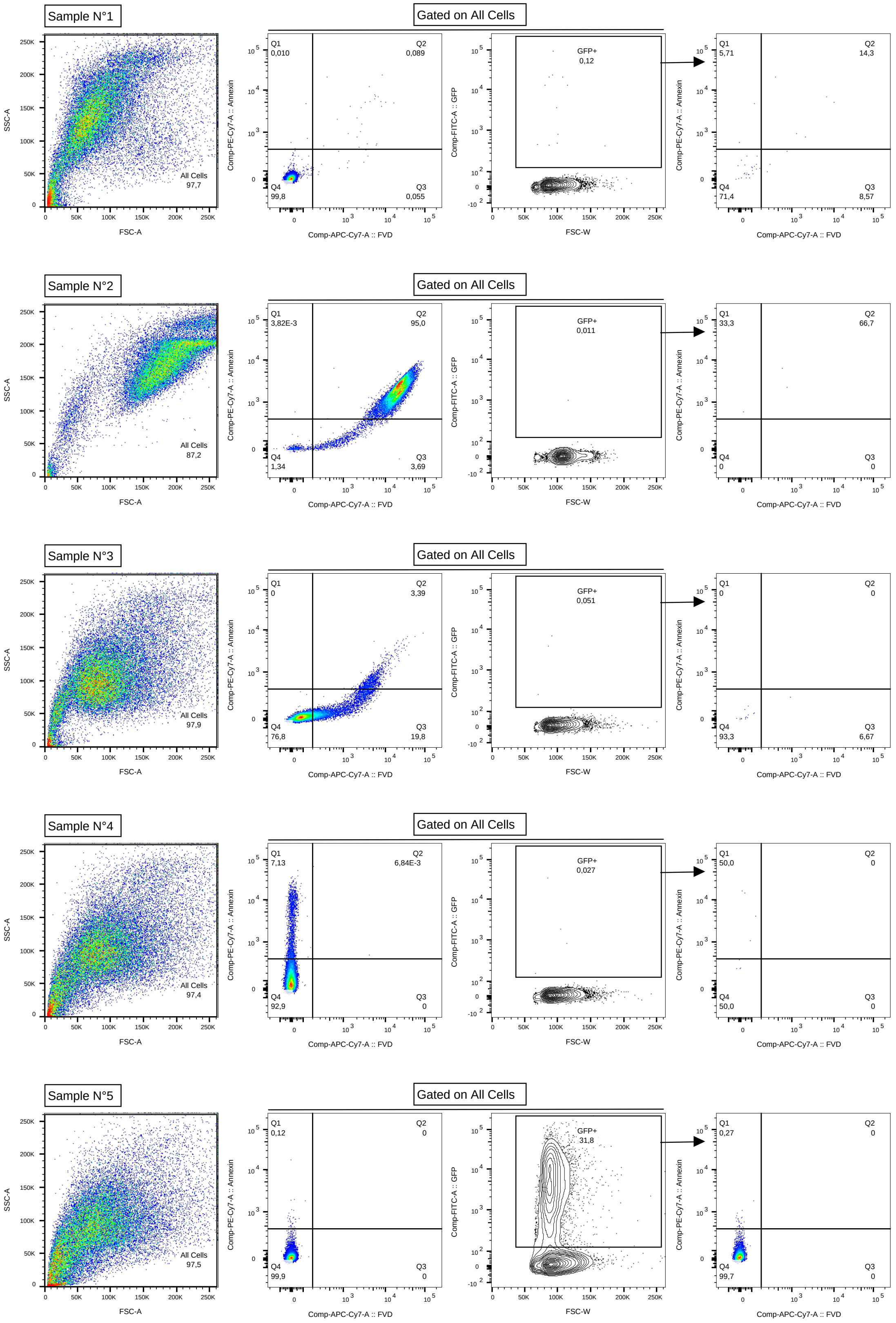

Sample N°1
Gated on All Cells
All Cells 97,7
Q1 0,010
Q2 0,089
Q3 0,055
Q4 99,8
GFP+ 0,12
Q1 5,71
Q2 14,3
Q3 8,57
Q4 71,4
Sample N°2
Gated on All Cells
All Cells 87,2
Q1 3,82E-3
Q2 95,0
Q3 3,69
Q4 1,34
GFP+ 0,011
Q1 33,3
Q2 66,7
Q3 0
Q4 0
Sample N°3
Gated on All Cells
All Cells 97,9
Q1 0
Q2 3,39
Q3 19,8
Q4 76,8
GFP+ 0,051
Q1 0
Q2 0
Q3 6,67
Q4 93,3
Sample N°4
Gated on All Cells
All Cells 97,4
Q1 7,13
Q2 6,84E-3
Q3 0
Q4 92,9
GFP+ 0,027
Q1 50,0
Q2 0
Q3 0
Q4 50,0
Sample N°5
Gated on All Cells
All Cells 97,5
Q1 0,12
Q2 0
Q3 0
Q4 99,9
GFP+ 31,8
Q1 0,27
Q2 0
Q3 0
Q4 99,7
SSC-A
FSC-A
Comp-PE-Cy7-A :: Annexin
Comp-APC-Cy7-A :: FVD
Comp-FITC-A :: GFP
FSC-W

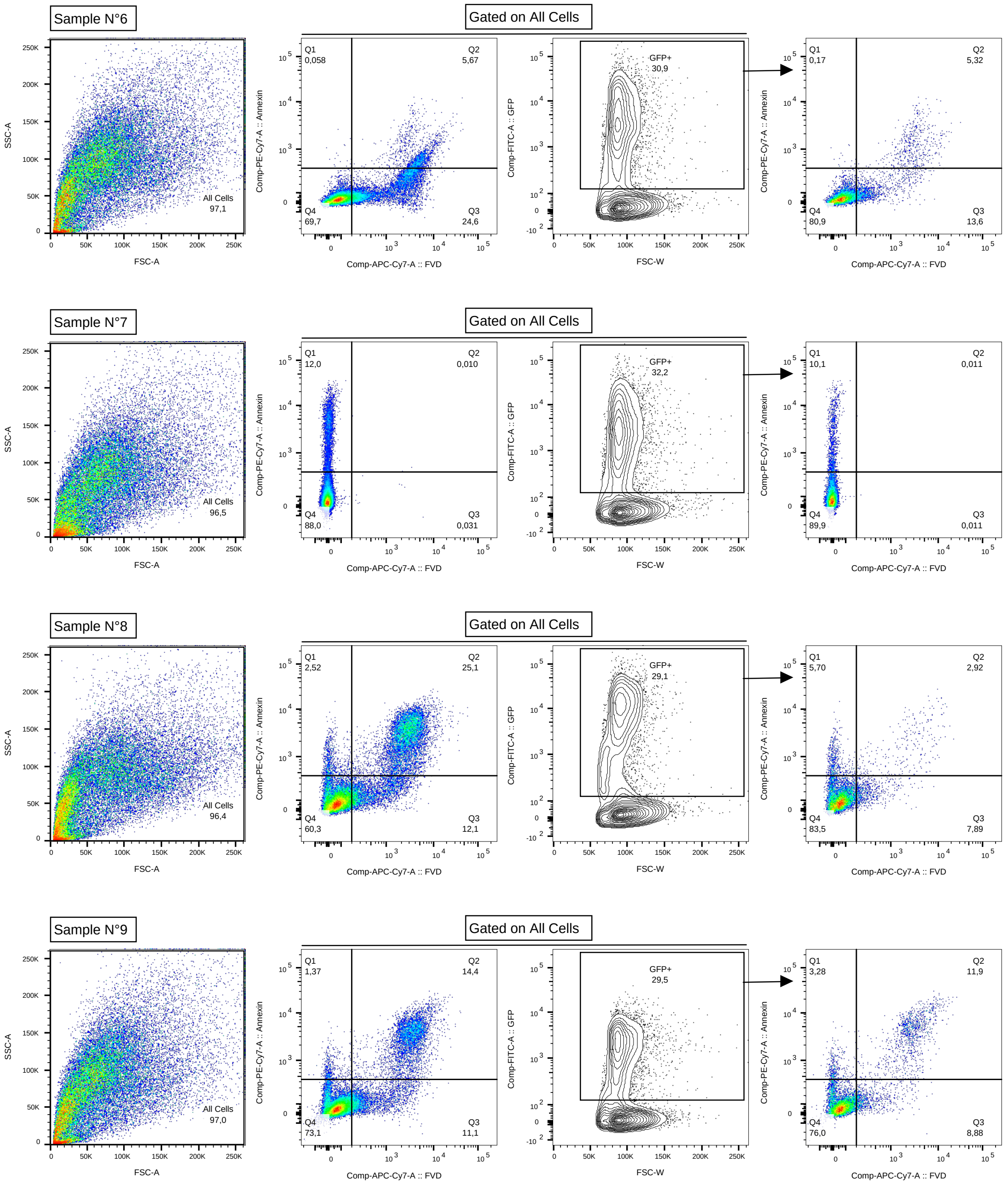

Sample N°6
Gated on All Cells
All Cells 97,1
Q1 0,058
Q2 5,67
Q3 24,6
Q4 69,7
GFP+ 30,9
Q1 0,17
Q2 5,32
Q3 13,6
Q4 80,9
Sample N°7
Gated on All Cells
All Cells 96,5
Q1 12,0
Q2 0,010
Q3 0,031
Q4 88,0
GFP+ 32,2
Q1 10,1
Q2 0,011
Q3 0,011
Q4 89,9
Sample N°8
Gated on All Cells
All Cells 96,4
Q1 2,52
Q2 25,1
Q3 12,1
Q4 60,3
GFP+ 29,1
Q1 5,70
Q2 2,92
Q3 7,89
Q4 83,5
Sample N°9
Gated on All Cells
All Cells 97,0
Q1 1,37
Q2 14,4
Q3 11,1
Q4 73,1
GFP+ 29,5
Q1 3,28
Q2 11,9
Q3 8,88
Q4 76,0
SSC-A
FSC-A
Comp-PE-Cy7-A :: Annexin
Comp-APC-Cy7-A :: FVD
Comp-FITC-A :: GFP
FSC-W

MIA PaCa 2

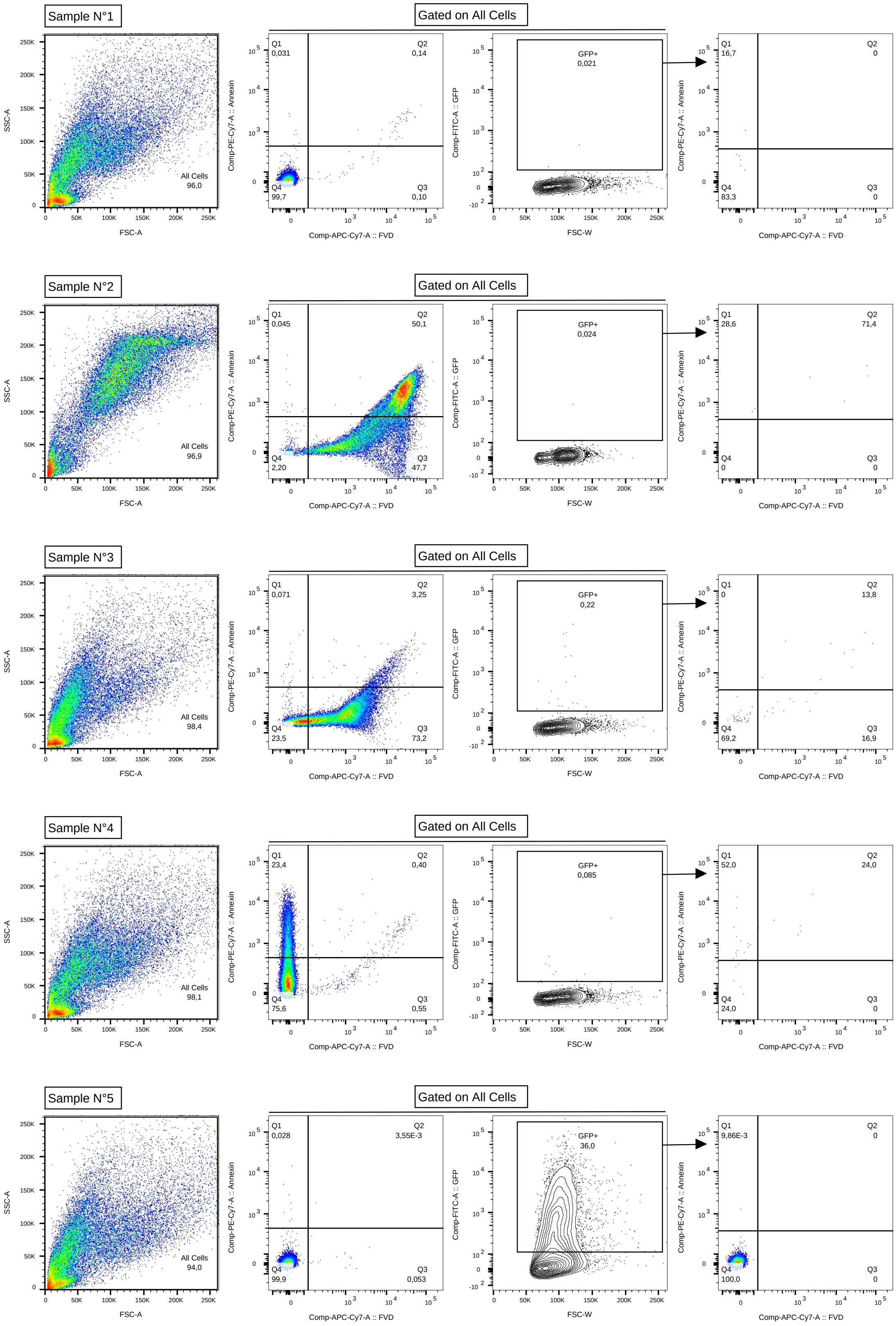
Sample N°1
Gated on All Cells
SSC-A
FSC-A
All Cells
96,0
Comp-PE-Cy7-A :: Annexin
Comp-APC-Cy7-A :: FVD
Q1
0,031
Q2
0,14
Q3
0,10
Q4
99,7
Comp-FITC-A :: GFP
FSC-W
GFP+
0,021
Q1
16,7
Q2
0
Q3
0
Q4
83,3
Sample N°2
Gated on All Cells
All Cells
96,9
Q1
0,045
Q2
50,1
Q3
47,7
Q4
2,20
GFP+
0,024
Q1
28,6
Q2
71,4
Q3
0
Q4
0
Sample N°3
Gated on All Cells
All Cells
98,4
Q1
0,071
Q2
3,25
Q3
73,2
Q4
23,5
GFP+
0,22
Q1
0
Q2
13,8
Q3
16,9
Q4
69,2
Sample N°4
Gated on All Cells
All Cells
98,1
Q1
23,4
Q2
0,40
Q3
0,55
Q4
75,6
GFP+
0,085
Q1
52,0
Q2
24,0
Q3
0
Q4
24,0
Sample N°5
Gated on All Cells
All Cells
94,0
Q1
0,028
Q2
3,55E-3
Q3
0,053
Q4
99,9
GFP+
36,0
Q1
9,86E-3
Q2
0
Q3
0
Q4
100,0

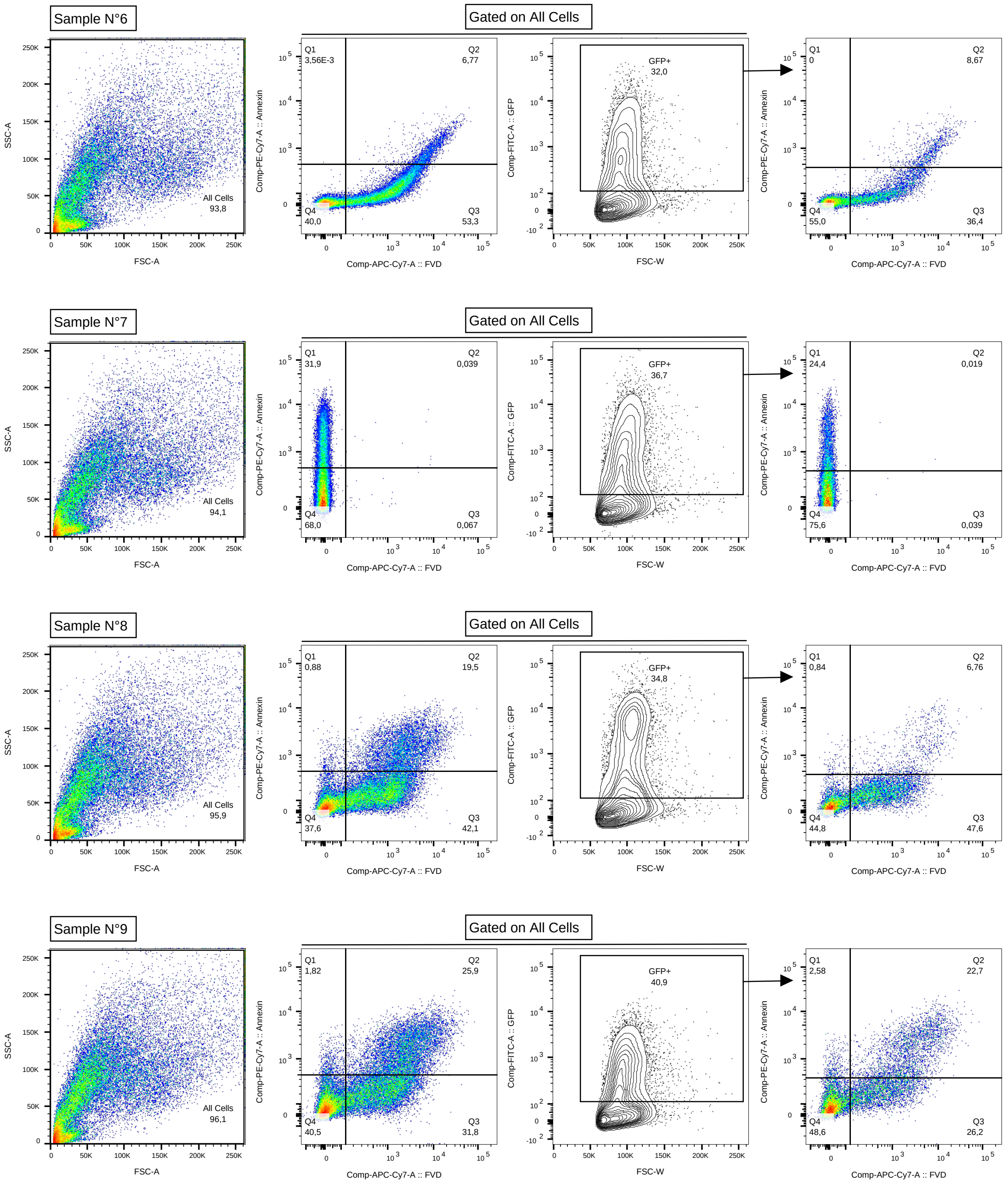

Sample N°6
Gated on All Cells
All Cells 93,8
Q1 3,56E-3
Q2 6,77
Q3 53,3
Q4 40,0
GFP+ 32,0
Q1 0
Q2 8,67
Q3 36,4
Q4 55,0
Sample N°7
Gated on All Cells
All Cells 94,1
Q1 31,9
Q2 0,039
Q3 0,067
Q4 68,0
GFP+ 36,7
Q1 24,4
Q2 0,019
Q3 0,039
Q4 75,6
Sample N°8
Gated on All Cells
All Cells 95,9
Q1 0,88
Q2 19,5
Q3 42,1
Q4 37,6
GFP+ 34,8
Q1 0,84
Q2 6,76
Q3 47,6
Q4 44,8
Sample N°9
Gated on All Cells
All Cells 96,1
Q1 1,82
Q2 25,9
Q3 31,8
Q4 40,5
GFP+ 40,9
Q1 2,58
Q2 22,7
Q3 26,2
Q4 48,6
SSC-A
FSC-A
Comp-PE-Cy7-A :: Annexin
Comp-APC-Cy7-A :: FVD
Comp-FITC-A :: GFP
FSC-W

H1975

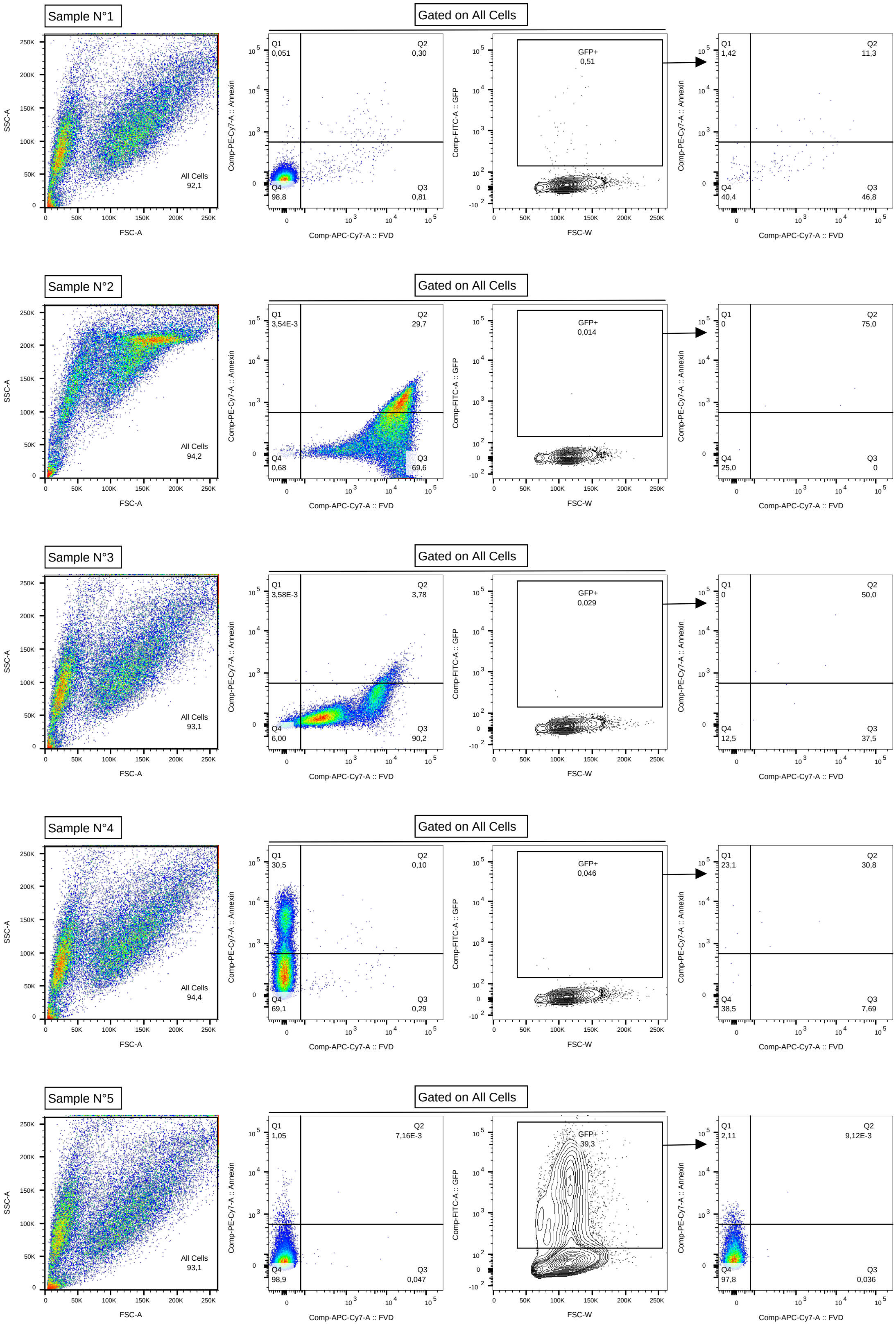

Sample N°1
Gated on All Cells
All Cells 92,1
Q1 0,051
Q2 0,30
Q4 98,8
Q3 0,81
GFP+ 0,51
Q1 1,42
Q2 11,3
Q4 40,4
Q3 46,8
Sample N°2
Gated on All Cells
All Cells 94,2
Q1 3,54E-3
Q2 29,7
Q4 0,68
Q3 69,6
GFP+ 0,014
Q1 0
Q2 75,0
Q4 25,0
Q3 0
Sample N°3
Gated on All Cells
All Cells 93,1
Q1 3,58E-3
Q2 3,78
Q4 6,00
Q3 90,2
GFP+ 0,029
Q1 0
Q2 50,0
Q4 12,5
Q3 37,5
Sample N°4
Gated on All Cells
All Cells 94,4
Q1 30,5
Q2 0,10
Q4 69,1
Q3 0,29
GFP+ 0,046
Q1 23,1
Q2 30,8
Q4 38,5
Q3 7,69
Sample N°5
Gated on All Cells
All Cells 93,1
Q1 1,05
Q2 7,16E-3
Q4 98,9
Q3 0,047
GFP+ 39,3
Q1 2,11
Q2 9,12E-3
Q4 97,8
Q3 0,036
SSC-A
FSC-A
Comp-PE-Cy7-A :: Annexin
Comp-APC-Cy7-A :: FVD
Comp-FITC-A :: GFP
FSC-W

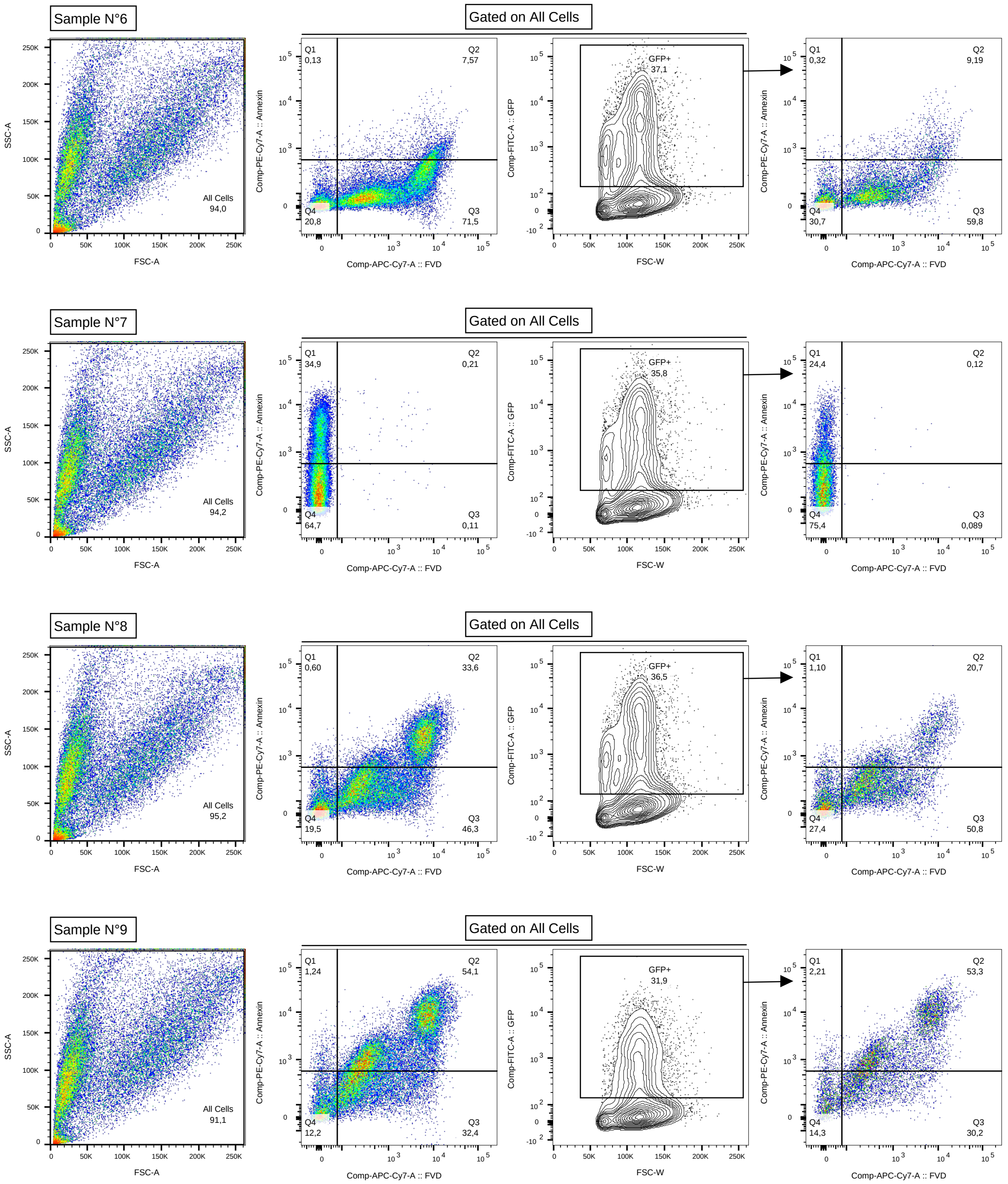

Sample N°6
Gated on All Cells
All Cells 94,0
Q1 0,13
Q2 7,57
Q3 71,5
Q4 20,8
GFP+ 37,1
Q1 0,32
Q2 9,19
Q3 59,8
Q4 30,7
Sample N°7
Gated on All Cells
All Cells 94,2
Q1 34,9
Q2 0,21
Q3 0,11
Q4 64,7
GFP+ 35,8
Q1 24,4
Q2 0,12
Q3 0,089
Q4 75,4
Sample N°8
Gated on All Cells
All Cells 95,2
Q1 0,60
Q2 33,6
Q3 46,3
Q4 19,5
GFP+ 36,5
Q1 1,10
Q2 20,7
Q3 50,8
Q4 27,4
Sample N°9
Gated on All Cells
All Cells 91,1
Q1 1,24
Q2 54,1
Q3 32,4
Q4 12,2
GFP+ 31,9
Q1 2,21
Q2 53,3
Q3 30,2
Q4 14,3
SSC-A
FSC-A
Comp-PE-Cy7-A :: Annexin
Comp-APC-Cy7-A :: FVD
Comp-FITC-A :: GFP
FSC-W

Table S2. Tumors selection workflow

	Mutations count in *TP53* DBD						
	R175	R213	R248	R273	R282	Selected tumors	Mutation counts per tumor type
**Adrenocortical cancer**	0	0	0	1	0		1
**Bladder urothelial carcinoma**	3	1	20	7	2	X	33
**Brain lower grade glioma**	8	1	11	66	6	X	92
**Breast invasive carcinoma**	15	0	7	17	3	X	42
**Cholangiocarcinoma**	0	0	1	0	0		1
**Colon adenocarcinoma**	17	2	19	16	10	X	64
**Diffuse large B-cell lymphoma**	0	0	1	1	0		2
**Esophageal carcinoma**	9	0	13	10	6	X	38
**Glioblastoma multiforme**	3	0	4	4	1	X	12
**Head & neck squamous cell carcinoma**	6	1	16	14	10	X	47
**Kidney chromophobe**	0	0	0	0	1		1
**Kidney clear cell carcinoma**	0	1	1	0	0		2
**Liver hepatocellular carcinoma**	1	2	3	2	0	X	8
**Lung adenocarcinoma**	1	0	5	9	2	X	17
**Lung squamous cell carcinoma**	8	2	10	15	5	X	40
**Mesothelioma**	0	0	0	2	0		2
**Pancreatic adenocarcinoma**	4	0	8	5	3	X	20
**Prostate adenocarcinoma**	2	0	5	1	2	X	10
**Rectum adenocarcinoma**	7	1	7	7	8	X	30
**Sarcoma**	5	1	4	1	0		11
**Stomach adenocarcinoma**	12	0	7	17	6	X	42
**Thymoma**	0	0	1	1	0		2
**Uterine carcinosarcoma**	2	0	7	4	0	X	13
**Uterine corpus endometrioid carcinoma**	9	0	10	14	6	X	39
**Total counts**	112	12	160	214	71		569

Table S1. Primer sequences used in the RT-qPCR assays.

Target	Primer name	Sequence (5’→3’)
β-*actin*	β-actin H F<br>β-actin H R	CACTCTTCCAGCCTTCCT<br>CAGGTCTTTGCGGATGTC
Myc	Myc H F<br>Myc H R	CACACATCAGCACAACTACG<br>CTGTCCAACTTGACCCTCTT
Tp63	TP63 H F<br>TP63 H R	CGTGTCCTTCCAGCAGTC<br>GGCAGTAGAGTTTCTTCAGTTCA
