# Supplementary material for: HOPS/TMUB1 Enhances Apoptosis in TP53 Mutation-Independent Setting in Human Cancers
Source: Int J Mol Sci. 2024 Apr 23;25(9):4600. doi: 10.3390/ijms25094600 (PMC11083489; doi:10.3390/ijms25094600)
Supplement: Supplementary file 1 [file ijms-25-04600-s001.zip › Table S1.pdf]

Table S1. Primer sequences used in the RT-qPCR assays.

Target	Primer name	Sequence (5'→3')
β-*actin*	β-actin H F<br>β-actin H R	CACTCTTCCAGCCTTCCT<br>CAGGTCTTTGCGGATGTC
Myc	Myc H F<br>Myc H R	CACACATCAGCACAACTACG<br>CTGTCCAACTTGACCCTCTT
Tp63	TP63 H F<br>TP63 H R	CGTGTCCTTCCAGCAGTC<br>GGCAGTAGAGTTTCTTCAGTTCA
