# Supplementary material for: HOPS/TMUB1 Enhances Apoptosis in TP53 Mutation-Independent Setting in Human Cancers
Source: Int J Mol Sci. 2024 Apr 23;25(9):4600. doi: 10.3390/ijms25094600 (PMC11083489; doi:10.3390/ijms25094600)
Supplement: Supplementary file 1 [file ijms-25-04600-s001.zip › Table S2.pdf]

Table S2. Tumors selection workflow

	Mutations count in *TP53* DBD						
	R175	R213	R248	R273	R282	Selected tumors	Mutation counts per tumor type
**Adrenocortical cancer**	0	0	0	1	0		1
**Bladder urothelial carcinoma**	3	1	20	7	2	X	33
**Brain lower grade glioma**	8	1	11	66	6	X	92
**Breast invasive carcinoma**	15	0	7	17	3	X	42
**Cholangiocarcinoma**	0	0	1	0	0		1
**Colon adenocarcinoma**	17	2	19	16	10	X	64
**Diffuse large B-cell lymphoma**	0	0	1	1	0		2
**Esophageal carcinoma**	9	0	13	10	6	X	38
**Glioblastoma multiforme**	3	0	4	4	1	X	12
**Head & neck squamous cell carcinoma**	6	1	16	14	10	X	47
**Kidney chromophobe**	0	0	0	0	1		1
**Kidney clear cell carcinoma**	0	1	1	0	0		2
**Liver hepatocellular carcinoma**	1	2	3	2	0	X	8
**Lung adenocarcinoma**	1	0	5	9	2	X	17
**Lung squamous cell carcinoma**	8	2	10	15	5	X	40
**Mesothelioma**	0	0	0	2	0		2
**Pancreatic adenocarcinoma**	4	0	8	5	3	X	20
**Prostate adenocarcinoma**	2	0	5	1	2	X	10
**Rectum adenocarcinoma**	7	1	7	7	8	X	30
**Sarcoma**	5	1	4	1	0		11
**Stomach adenocarcinoma**	12	0	7	17	6	X	42
**Thymoma**	0	0	1	1	0		2
**Uterine carcinosarcoma**	2	0	7	4	0	X	13
**Uterine corpus endometrioid carcinoma**	9	0	10	14	6	X	39
**Total counts**	112	12	160	214	71		569
